# Self-Assembly of Antiferromagnetically-Coupled Copper(II) Supramolecular Architectures with Diverse Structural Complexities

**DOI:** 10.3390/molecules25235549

**Published:** 2020-11-26

**Authors:** Santokh S. Tandon, Scott D. Bunge, Neil Patel, Esther C. Wang, Laurence K. Thompson

**Affiliations:** 1 Department of Chemistry and Biochemistry, Kent State University-Salem Campus, Salem, OH 44460, USA; 2 Department of Chemistry and Biochemistry, Kent State University, Kent, OH 44242, USA; sbunge@kent.edu (S.D.B.); neilnpatel89@gmail.com (N.P.); ewang2@kent.edu (E.C.W.); 3 Department of Chemistry, Memorial University, St. John’s Newfoundland, NL A1B 3X7, Canada; laurie.thomp@gmail.com

**Keywords:** self-assembly of supramolecular architectures, antiferromagnetically-coupled copper(II) complexes, magneto-structural correlations, phenolate and azido bridged copper(II) complexes

## Abstract

The self-assembly of 2,6-diformyl-4-methylphenol (**DFMP**) and 1-amino-2-propanol (**AP**)/2-amino-1,3-propanediol (**APD**) in the presence of copper(II) ions results in the formation of six new supramolecular architectures containing two versatile double Schiff base ligands (H_3_L and H_5_L1) with one-, two-, or three-dimensional structures involving diverse nuclearities: tetranuclear [Cu_4_(HL^2−^)_2_(N_3_)_4_]·4CH_3_OH·56H_2_O (**1**) and [Cu_4_(L^3−^)_2_(OH)_2_(H_2_O)_2_] (**2**), dinuclear [Cu_2_(H_3_L1^2−^)(N_3_)(H_2_O)(NO_3_)] (**3**), polynuclear {[Cu_2_(H_3_L1^2−^)(H_2_O)(BF_4_)(N_3_)]·H_2_O}_n_ (**4**), heptanuclear [Cu_7_(H_3_L1^2−^)_2_(O)_2_(C_6_H_5_CO_2_)_6_]·6CH_3_OH·44H_2_O (**5**), and decanuclear [Cu_10_(H_3_L1^2−^)_4_(O)_2_(OH)**_2_**(C_6_H_5_CO_2_)_4_] (C_6_H_5_CO_2_)_2_·20H_2_O (**6**). X-ray studies have revealed that the basic building block in **1**, **3**, and **4** is comprised of two copper centers bridged through one μ-phenolate oxygen atom from HL^2−^ or H_3_L1^2−^, and one μ-1,1-azido (N_3_^−^) ion and in **2**, **5**, and **6** by μ-phenoxide oxygen of L^3−^ or H_3_L1^2−^ and μ-O^2−^ or μ_3_-O^2−^ ions. H-bonding involving coordinated/uncoordinated hydroxy groups of the ligands generates fascinating supramolecular architectures with 1D-single chains (**1** and **6**), 2D-sheets (**3**), and 3D-structures (**4**). In **5**, benzoate ions display four different coordination modes, which, in our opinion, is unprecedented and constitutes a new discovery. In **1**, **3**, and **5,** Cu(II) ions in [Cu_2_] units are antiferromagnetically coupled, with J ranging from −177 to −278 cm^−1^.

## 1. Introduction

Self-assembly processes use simple building blocks in biological systems for the construction of symmetrical complex supramolecular biomolecules such as proteins, lipoproteins, DNA, glycoproteins, enzymes etc. [1,2,3]. Inspired by nature, in the last few decades, chemists have used the self-assembly methodology [4,5,6] to successfully generate a variety of supramolecular architectures including organic materials [7,8,9], metalacyclic polygons and polyhedrons [10], and nanoscale systems [11,12] with desirable sizes, shapes, and functions. The self-assembly technique, which utilizes a variety of cooperative and noncovalent interactions such as hydrogen bonding, strong electrostatic, and van der Walls forces, π-π stacking, hydrophobic, hydrophilic, and metal-ligand interactions etc., has many advantages over the stepwise synthesis of large supramolecular assemblies. In these processes, the formation of the desired products from the building blocks occur spontaneously and efficiently in one pot. For the spontaneous self-assembly of supramolecular coordination complexes including 1D-, 2D-, and 3D-network structures and grids, appropriate precursor building blocks are reacted in the presence of metal ions as templates [13,14,15,16,17,18,19,20,21,22,23].

We successfully used the self-assembly process to produce spin-coupled coordination complexes of transition metal ions with macrocyclic [23,24,25,26,27,28,29,30,31,32,33,34,35,36,37,38] and noncyclic [39,40,41,42,43] Schiff base ligands to get a deeper insight into magneto-structural relationships, to understand the role of metal ions on the self-assembly and structural complexities of assemblies, and the effects of the anions on the formation and coordination abilities of the Schiff-base ligands. Our interest in this area stemmed from the implications of transition metal complexes in homogeneous catalysis [44,45,46] as enzyme models [47,48,49,50], and their potential applications in magnetic materials [51,52,53,54,55]. In the last three decades, many spin-coupled coordination complexes exhibiting ferromagnetic and antiferromagnetic behavior have been designed, synthesized and structurally characterized, and magneto-structural relationships have been investigated [56,57,58,59,60,61,62,63,64,65,66,67,68,69,70,71,72,73,74,75,76,77,78,79,80,81,82,83,84,85,86,87,88]. In spin coupled coordination clusters, the bridging ligands between metal centers play a central role on the nature and the magnitude of the magnetic spin exchange interactions. In addition to organic ligands, a variety of doubly, triply, or quadruply bridging anions like N_3_^−^, RO^−^ (R= H, C_6_H_5_, CH_3_ etc.), N(CN)_2_^−^, CN^−^, NCS^−^, O^2−^, RCO_2_^−^ (R = C_6_H_5_, CH_3_) are used often to generate extended network structures [89,90,91,92,93,94]. Due to the flexi-dentate nature and diverse coordination modes, hydroxy and azido are the most versatile anions for providing intra/interdimer bridges to generate supramolecular clusters and to propagate spin-exchange interactions between the metal centers [40,42,53,95,96,97,98,99,100,101,102,103,104].

In our earlier communications, we reported the self-assembly of 2,6-diformyl-4-alkyl(R)phenol (R = CH_3_, **DFMP** and R = C(CH_3_)_3_, **DFTBP**) with diamine/diamino alcohols in the presence of Lewis acids (H^+^/transition metal ions) as a templating agent to generate metal free macrocycles [27] and coordination complexes of macrocyclic ligands with different nuclearities ranging from dinuclear [28,30,32,34,36] to tetranuclear/dimeric octanuclear [24,25,26,29,30,32,37], and hexanuclear/dimeric dodecanuclear clusters in which a single macrocycle incorporates six metal ions in a distorted boat-shaped ‘benzene-like’ array with three hydroxide ions in the central cavity [31,35], heptanuclear/dimeric tetradecanuclear body centered clusters with Cu^2+^ ion in the center of macrocyclic cavity [38], and dimeric dodecanuclear supramolecular metallo-clusters in which the central BO_3_^3−^ species is linked to six copper(II) ions held together by a single macrocyclic ligand through three μ1,1-O(BO_3_^3−^) and three μ1,3-O(BO_3_^3−^) bridges [23]. In continuation of our interest in the self-assembly of spin coupled transition metal complexes of cyclic/noncyclic Schiff-base ligands and our investigation of magneto-structural relationships, the effects of various anions and the transition metal ions on the self-assembly of supramolecular architectures with different nuclearities and structural complexities, and the formation and the coordination abilities of Schiff-base ligands, we have undertaken a systematic approach of reacting **DFMP** and various hydroxy-amines incorporating one, two, and three hydroxy groups. The self-assembly of **DFMP** and 2-aminoethanol in the presence of Co^2+^/Ni^2+^/NaN_3_ generates ferromagnetically-coupled tetranuclear and hexanuclear azide-bridged 1D single-chain coordination polymers [40], whereas in the presence of Cu^2+^/NaN_3_ antiferromagnetically-coupled copper coordination, polymers based on single-chain or sheet structures involving dinuclear and tetranuclear copper(II) units are produced [41]. One pot self-assembly reactions between **DFMF** and tris(hydroxymethyl)aminomethane (**THMAM**) in the presence of copper(II)/nickel(II)/NaC_6_H_5_CO_2_/(NaN_3_) produce an antiferromagnetically-coupled Cu(II) coordination polymer consisting of repeating pentanuclear units with a novel double-stranded ladder-like structure in which [Cu(N_3_)_4_]^2−^ anions link single chains comprised of dinuclear cationic [Cu_2_(H_5_L3^2−^)(μ-N_3_)]^+^subunits, forming a 3D structure of interconnected ladders through H bonding [39,42] and ferromagnetically-coupled hexanuclear nickel(II) clusters [42]. One-pot self-assembly reactions of **DFMF** with 1-amino-2-propanol (**AP**) and 2-amino-1,3-propanediol (**APD**) in the presence of nickel(II)/NaN_3_ give antiferromagnetically-coupled tetranuclear coordination complexes of nickel(II) with incomplete double cubane structural cores which form 1D-single chains, 2D-sheets, and 3D structures through a network of H-bonding [43]. The coordination versatility of the Schiff-base ligands H_3_L (2,6-bis-{(2-hydroxypropylimino)methyl}-4-methylphenol), potentially pentadentate (N_2_O_3_), trianionic ligand) and H_5_L1 (2,2′-[(2-hydroxy-5-methyl-1,3-phenylene) bis (methylidynenitrilo)]-1,3-propanediol, potentially heptadentate (N_2_O_5_), pentaanionic ligand) towards nickel(II) ions [43] has prompted us to explore the coordination chemistry of these ligands towards copper(II) ions. In this report, the synthesis, crystal structures, and magnetic properties of six new copper(II) coordination complexes of two double Schiff-base ligands H_3_L and H_5_L1 with diverse nuclearities (Cu_2_, Cu_4_, Cu_7_, Cu_10_, Cu_n_) are presented. Variable temperature magnetic studies performed on three complexes (**1**, **3**, and **5**) revealed that the magnetic exchange interactions within dinuclear units are dominated by strong antiferromagnetic coupling (J ranging from −177 to −278 cm^−1^).

## 2. Results and Discussion

### 2.1. Synthesis of the Complexes

**Figure 1:**
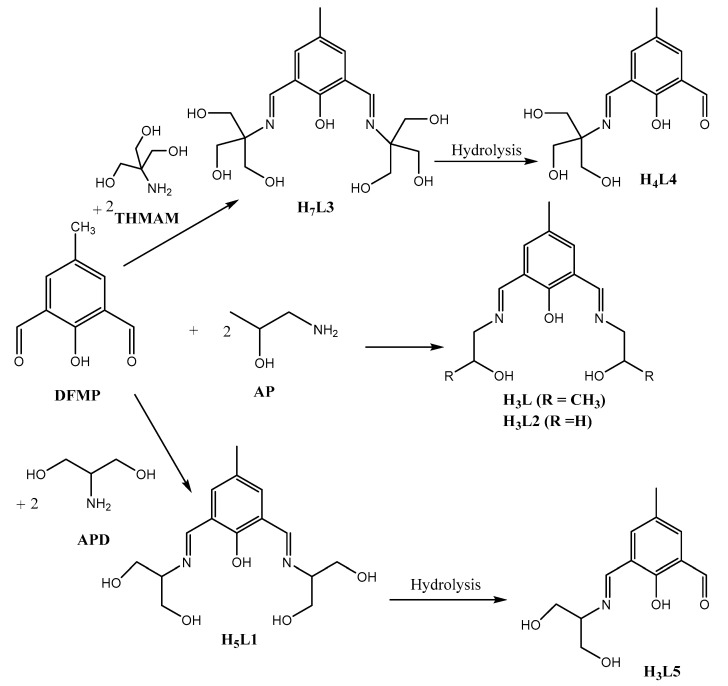
Structures of the Schiff base ligands. **DFMP** (2,6-diformyl-4-methylphenol), **APD** (2-amino-1,3-propanediol), **AP** (1-amino-2-propanol), and **THMAM** (tris(hydroxymethyl) amino methane).

Herein, we report the self-assembly, structural characterization, and magnetic studies of six new copper(II) complexes of two very versatile double Schiff base ligands (H_3_L and H_5_L1) with a high degree of conformational flexibility. Previously [43], we reported the H-bonding directed self-assembly of ferromagnetically-coupled tetranuclear nickel(II) complexes of Schiff-base ligands H_3_L and H_5_L1 with one-, two- and three dimensional structural complexities. Reactions of **DFMP** with 1-amino-2-propanol (**AP**) and 2-amino-1,3-propanediol (**APD**) in the presence of copper(II) salts, CuX_2_ (X = CH_3_CO_2_^−^, NO_3_, Cl, ClO_4_, BF_4_^−^)/NaN_3_/TEA, under varied conditions in one-pot, self-assembly produced dinuclear (**3**), tetranuclear (**1** and **2**), heptanuclear (**5**), decanuclear (**6**), and polynuclear (**4**) copper(II) complexes of H_3_L and H_5_L1 ligands. These complexes grow into beautiful 1D-single chains, 2D-sheets, or 3D structures through network of H-bonding. Previously [41,42,43], we observed that the nature of the anions and the metal ions had remarkable effects on the self-assembly of polynuclear supramolecular clusters, on the structural complexities, and the coordination abilities of the ligands. In the presence of Ni(II) ions [42,43], initially formed double Schiff-base ligands (H_5_L1 and H_7_L3) undergo metal catalyzed partial hydrolysis of the double Schiff-base ligands to produce tetranuclear [Ni_4_(HL5^−2^)_2_(APD^−1^)_2_](ClO_4_)_2_ and hexanuclear [Ni_6_(H_3_L4^−1^)_2_(THMAM^2−^)_2_(µ-N_3_)_4_(CH_3_CO_2_)_2_] and [Ni_6_(H_3_L4^−1^)_2_(THMAM^2−^)_2_(µ-N_3_)_4_(C_6_H_2_CO_2_)_2_] complexes of mono Schiff-base ligands H_3_L5 and H_4_L4, respectively (Figure 1). Complexes **1**, **3**, and **4** exhibit the same type of [Cu_2_] basic building block cemented by the phenoxide O-atom of the Schiff base and µ-1,1-azido bridge which dimerizes or polymerizes producing tetranuclear or supramolecular architectures through a variety of alkoxide, methoxide, oxide, and azido bridges. In complexes **1** and **2**, H_3_L holds two copper centers in close proximity forming dinuclear units in which Cu(II) ions are bridged by phenoxide oxygen and µ-1,1-N_3_ (**1**) /µ_3_-OH^−^ (**2**) bridges. The dinuclear units dimerize through two interdimer µ-1,1-N_3_ bridges (**1**) or through two µ_3_-OH^−^ and two µ_3_-PhO^−^ (intra/interdimer) bridges that form neutral centrosymmetric tetranuclear complex (**2**). In complexes **3** and **4**, H_5_L1 holds two Cu(II) centers via phenoxide oxygen and µ-1,1-N_3_ bridges that form dinuclear units which grow into 2D-Sheets (**3**)/1D-single chains (**4**), which are crosslinked to generate 3D-structures through a network of strong H-bonding. In complexes **5** and **6**, H_5_L1 holds two Cu(II) ions through phenoxide oxygen and µ_3_-O^2−^/µ-OH^−^ bridges to form dinuclear units which are interconnected through benzoate/µ_3_-O^2−^/µ-OH^−^ bridges to generate heptanuclear (**5**) and decanuclear (**6**) complexes, which are relatively rare nuclearities. In **5**, benzoate ions display 4 different coordination modes which, in our opinion, is unprecedented and constitutes a first report. In complex **6**, decanuclear units are interconnected through H-bonds producing a supramolecular 1D-single chains structure.

### 2.2. Description of Structures

The Cu-N and Cu-O distances in the equatorial plane of all the complexes (**1**–**6**) reported in this paper fall in the ranges 1.888(13)–2.001(7) Å and 1.874(10)–2.038(4) Å (data Tables S1–S6), respectively, like other reported complexes with similar Schiff-base ligands [40,41,42,105,106,107,108]. The long axial Cu–O distances lie in the range 2.337(2)–2.79(7) Å. The Cu–Cu separation in dinuclear units (Cu_2_) of these complexes lies in the range 2.92(5)–3.09(2) Å, similar to other dinuclear units in copper, cobalt and nickel complexes with Schiff-base ligands [40,41,42,105,106,107,108]. The Cu–Cu distance between dinuclear units lies in the range 2.865(13)–3.58(1) Å, which, in some cases, is significantly longer than the intermetallic separation within dinuclear units, and is similar to that reported in similar tetranuclear copper(II) complexes [41].

#### 2.2.1. [Cu_4_(HL^2−^)_2_(N_3_)_4_]·4CH_3_OH·56H_2_O (**1**)

**Figure 2:**
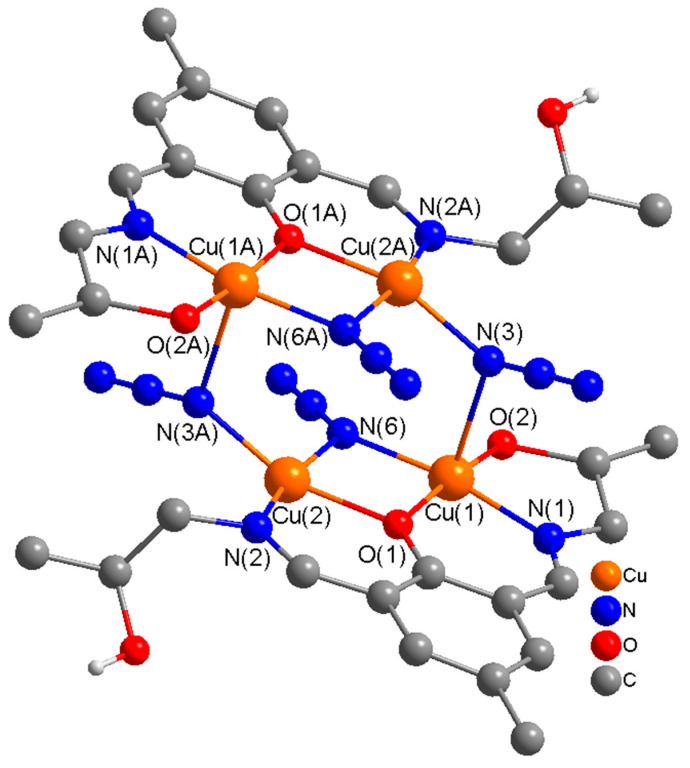
Molecular structure of a neutral centrosymmetric tetranuclear unit [Cu_4_(HL^2−^)_2_(N_3_)_4_] (**1**) with numbering of atoms in the coordination environment. H atoms and solvent molecules are omitted for clarity. Atoms with A in their labels are symmetry generated.

**Figure 3:**
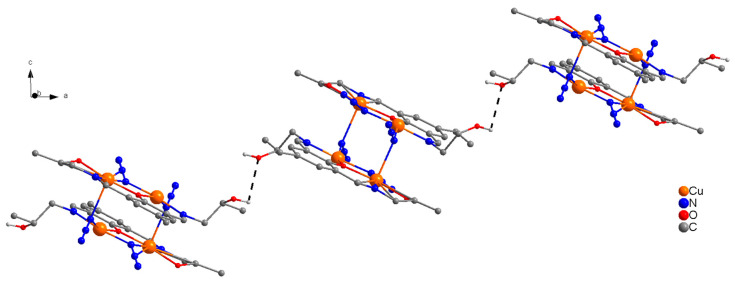
Perspective view of the crystal packing of **1** as seen along the *a*-axis generating 1D-single chains via protonated uncoordinated hydroxy groups in the side arm of the ligands.

The molecular structure of centrosymmetric complex **1** is shown in Figure 2, together with relevant atomic labeling. Important bond distances and angles are listed in Table S1. In complex **1**, H_3_L acts as a tetradentate (N_2_O_2_) dianionic ligand (HL^2−^), binding through two imine nitrogen atoms, a deprotonated alkoxide oxygen in the side arm of the ligand, and a deprotonated phenoxide oxygen, bridging two copper(II) ions into a dinuclear unit. The alkoxy group on one side of the Schiff-base ligand remains protonated and uncoordinated. In each dinuclear unit, two copper(II) ions are bridged through a phenoxide oxygen and a μ-1,1-N_3_ bridge. The link between [Cu_2_] pairs is established via two end-on (EO) μ-1,1-N_3_ bridges that form neutral centrosymmetric tetranuclear units, which are linked though remarkably strong H-bonds (2.688 Å) via protonated uncoordinated hydroxyl group (HO(3)) in the side arm of the ligand forming single chains along the *a*-axis. A perspective view of the polymeric single chains along the *a*-axis is presented in Figure 3.

The stereochemistry at Cu(1) in an asymmetric dinuclear unit can best be described as distorted square pyramidal with a phenoxide O, imine N, alkoxide O, and an azido nitrogen atom in the equatorial plane; and an azido nitrogen in the axial position (τ = 0.07); and a square planar geometry at Cu(2) (τ = 0.22) defined by phenoxide O, imine N, and two azido nitrogen atoms in the equatorial plane. (τ is a geometric parameter which is applicable to five coordinate structures as an index of the degree of trigonality). The sum of the angles in the basal plane of Cu(1) and Cu(2) are 359.4(3)° and 358.0(3)° respectively, indicating planar arrangements around these metal centers allowing effective overlap of the atomic orbitals for effective spin-exchange interactions between the metal centers. In a dinuclear unit, the bridge angles at the phenoxide oxygen (O(1)) and azido nitrogen (N(6)) are 101.88° and 102.4(3)° respectively. The sum of the angles around the phenoxide bridging O-atom, O(1), and an azide bridging N-atom, N(6) are 360.0(4)° and 356.8(5)° respectively, indicating fairly planar arrangements at these atoms to allow effective magnetic exchange interactions between the Cu(1) and Cu(2) ions in the dinuclear unit. The sum of the angles at N(3) (355.0(5)°), indicates some distortion from planarity. The bridge angle of 101.9(2)° at phenoxy oxygen, O(1) and of 102.4(3)° at the μ−1,1-N_3_ nitrogen, N(6), suggests an antiferromagnetic and a ferromagnetic interaction between copper centers within the dinuclear units [39,56].

#### 2.2.2. [Cu_4_(L^3−^)_2_(OH)_2_(H_2_O)_2_] (**2**)

**Figure 4:**
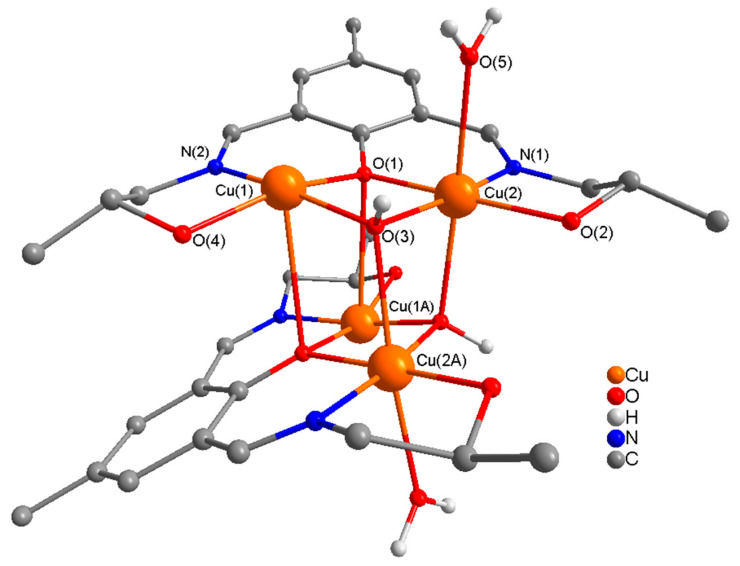
Molecular structure of the tetranuclear unit [Cu_4_(L^3−^)_2_(OH)_2_(H_2_O)_2_] (**2**) with numbering of relevant atoms in the coordination environment. H atoms and solvent molecules are omitted for clarity. Atoms with A in their labels are symmetry generated.

The molecular structure of centrosymmetric complex **2** is comprised of discreate neutral tetranuclear [Cu_4_(L^3−^)_2_(µ_3_-OH)_2_(H_2_O)_2_] units, and is shown in Figure 4, together with relevant atomic labeling. Important bond distances and angles are listed in Table S2. The coordination mode of H_3_L in **2** is quite different from that in complex **1**. In **2**, H_3_L utilizes its full coordination potential acting as a pentadentate (N_2_O_3_) trianionic (L^3−^) ligand by binding through two imine nitrogen, deprotonated phenoxy oxygen, and two deprotonated alkoxy oxygen atoms in the side arms. L^3−^ holds two Cu(II) ions in close proximity in a dinuclear unit bridged by two single atom bridges: a deprotonated phenoxy oxygen of the ligand, and a hydroxy bridge (µ_3_-OH^−^). The two dinuclear units are linked through two µ_3_-OH^−^ ions (Cu–O = 2.361 Å), which in addition to providing an intra-dinuclear bridge also act as an inter-dinuclear bridge, forming neutral tetranuclear units.

The geometry at Cu(1) in the dinuclear unit can best be described as distorted square pyramidal with phenoxide O, imine N, alkoxide O, and a µ_3_-hydroxy O atoms in the basal plane, and a phenoxy oxygen O(1A) in the axial plane (τ = 0.21); a distorted octahedral geometry at Cu(2) defined by phenoxide O, imine N, alkoxide O, and µ_3_-hydroxy O atoms in the equatorial plane and a µ_3_-hydroxy OA and a water O in the axial plane. The sum of the angles in the basal plane of Cu(1) and Cu(2) are 357.32(5)° and 359.98(15)° respectively, indicating almost planar arrangements around these metal centers. In a dinuclear unit the bridge angles at the phenoxide oxygen (O(1)) and µ_3_-OH^−^ (O(3)) are 98.02(14)° and 99.05(15)° respectively which are significantly smaller than observed in **1.** The sum of the angles around the phenoxide bridging O-atom, O(1) and µ_3_-OH^−^ bridging O-atom (O(3)) are 351.82(7)° and 301.19(14)° respectively, indicating a significant distortion from planarity and a strong pyramidal distortion respectively.

#### 2.2.3. [Cu_2_(H_3_L1^2−^)(N_3_)(H_2_O)(NO_3_)] (**3**)

**Figure 5:**
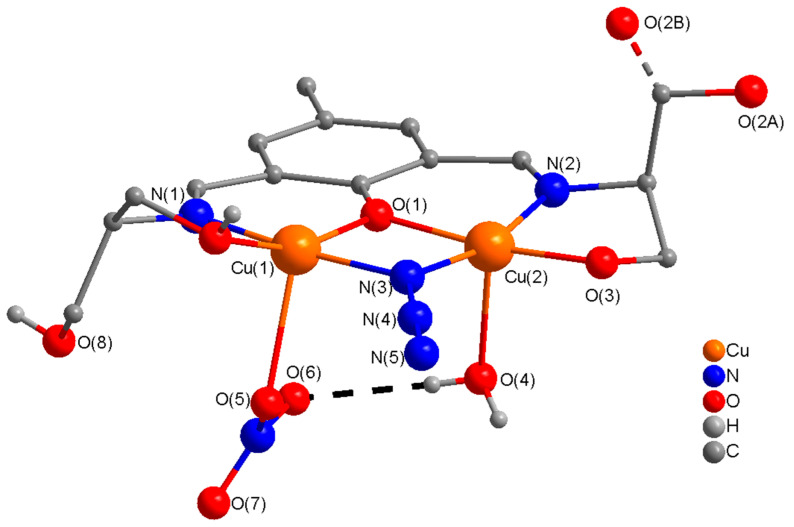
Molecular structure of a dinuclear unit [Cu_2_(H_3_L1^2−^)(H_2_O)(NO_3_)(N_3_)] (**3**) with relevant numbering. H-atoms are omitted for clarity.

**Figure 6:**
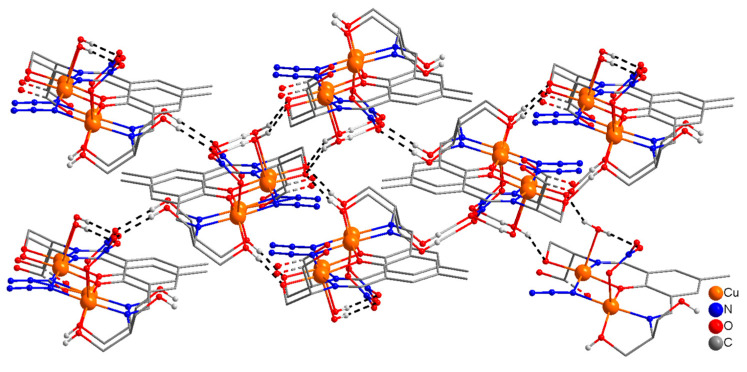
Perspective view of 3D-structure of **3**.

H_5_L1 is a potentially heptadentate penta-anionic double Schiff base ligand. Only one tetranuclear Ni^2+^ complex of this ligand has been reported [43]. In this publication, we are presenting the results of our investigation on the coordination versatility of this ligand towards copper(II) ions and the effect of the anions on the coordination ability of the ligand and the structural complexity. Reactions of copper(II) ions with H_5_L1 under varied conditions produce complexes of diverse nuclearities including dinuclear (**3**), heptanuclear (**5**), decanuclear (**6**), and polynuclear (**4**). In the dinuclear compound (**3**), H_3_L1^2−^ acts a pentadentate (N_2_O_3_) dianionic dinucleating ligand binding through phenoxide O, two imine N atoms, and two alkoxide O atoms in the side arms of the ligand. The second ethanol (–OH) group in the side arm of either side remains protonated and uncoordinated. On one side, oxygen atom (O(2A) & O(2B)) of ethanol group in the side arm is present at two positions with half-occupancy. In **3**, the copper centers are bridged by a phenoxide O(1) of H_3_L^2−^ and an end-on (EO) azido, µ-1,1-N_3_ bridge. The perspective view of **3** is shown in Figure 5. The dinuclear units grow into 2D-sheets along the *ab* or *ac*-axis (Figure S1), which are crosslinked along the *c* or *b*-axis to generate a very fascinating supramolecular 3D-structure through a symmetrical, reasonably strong network of H-bonding interactions (2.511-3.000 Å) involving two uncoordinated protonated (O(2) and O(9)), coordinated protonated (O(8)), and coordinate deprotonated (O(3)). ethanoate groups in the side arms of the ligand (H_3_L1^2−^), coordinated water (O(4)), coordinated azide ion (N(5)), and coordinated nitrate ion (O(5), O(6), O(7)): O(2)---O(5) = 2.783 Å, O(4)---O(6) = 2.896 Å, O(9)---O(7) = 2.848 Å, O(3)---O(4) = 2.773 Å, O(3)---O(8) = 2.511 Å, and O(2)---N(5) = 3.00 Å. (see Figure 6).

The relevant bond distances and angles are listed in Table S3. The stereochemistry at each copper(II) ion can best be described as a distorted square pyramidal (τ (Cu(1) = 0.22 and Cu(2) = 0.13). The coordination geometry at each copper(II) ion in the basal plane consists of a phenoxide O-atom (O(1)), an imine N-atom, (N(1)/N(2)), an alkoxide O-atom, (O(8)/O(3)), and an azido nitrogen atom (N(3)), and a relatively longer contact with oxygen atom (O(5)/O(4)) of the nitrate/water at the axial position. The sum of the angles in the basal plane of Cu(1) and Cu(2) are 359.08(8)° and 358.68(8)° respectively, indicating planar arrangements at these metal centers. In a dinuclear unit of **3**, the bridge angles at the phenoxide oxygen atom (O(1)) and the azido nitrogen atom (N(3)) are 100.80(8)° and 100.07(9)°, respectively. The sum of the angles at the oxygen atom of the phenoxide bridge, O(1), and the nitrogen atom of the azido bridge, N(3), are 359.85(13)° and 358.37(15)° respectively, indicating planar arrangements around O(1) and N(3) for effective spin-exchange interactions between copper centers of the dinuclear core. The Cu(1)-Cu(2) distance of 3.019 Å in a dinuclear unit is intermediate between **1** (3.092 Å) and **2** (2.967 Å), and similar to the distance found in other dinuclear copper complexes with similar ligands [48,49,58,59,60,61,62,63,64,65,66].

#### 2.2.4. {[Cu_2_(H_3_L1^2−^)(H_2_O)(BF_4_)(N_3_)]·H_2_O}_n_ (**4**)

**Figure 7:**
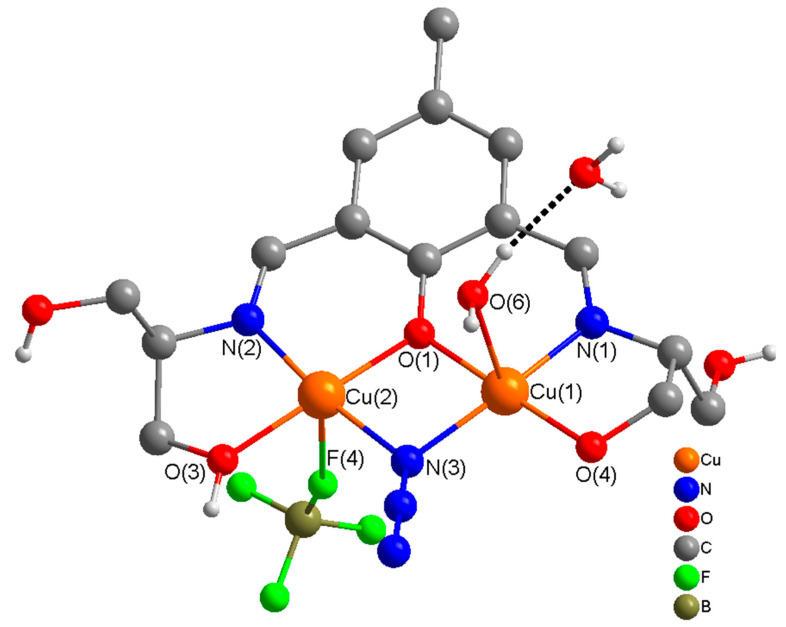
Molecular structure of a dinuclear unit [Cu_2_(H_3_L1^2−^)(H_2_O)(BF_4_)(N_3_)]·H_2_O in (**4**) with numbering of relevant atoms in the coordination environment. H-atoms are omitted for clarity.

**Figure 8:**
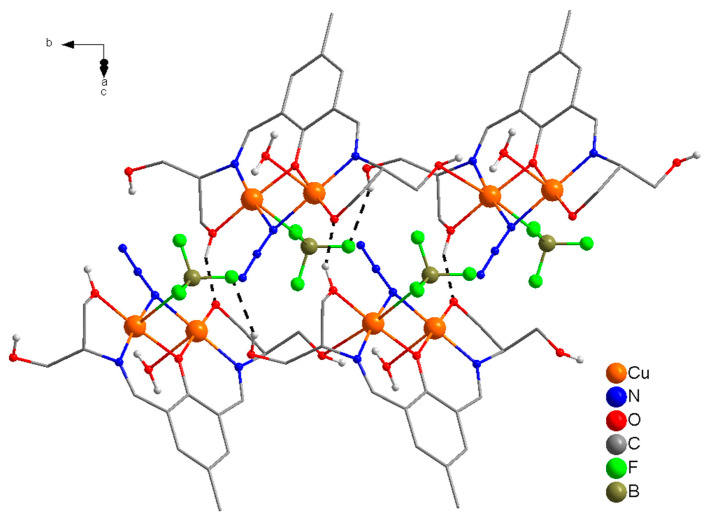
Perspective view of a portion of 3D supramolecular structure of **4**.

The molecular structure of a dinuclear unit in complex **4** is shown in Figure 7, together with relevant atomic labeling. Important bond distances and angles are listed in Table S4. The structure of compound **4** consists of polymeric one-dimensional single chains along the *b* axis, in which dinuclear [Cu_2_] units are linked via an oxygen atom (O(5)) of the ethanoate OH in the side arm of the ligand (Figure S2). These chains are cross linked via a strong network of intramolecular/intermolecular H-bonds [O(5)---O(6) = 2.739 Å, O(4)---O(7) = 2.885 Å, O(6)---O(7) = 2.788 Å, O(6)---O(7A) = 2.737 Å] involving the coordinated (O(4), (O5)) hydroxyl groups of ethanol in the side arms of the ligand and the coordinated (O(6))/uncoordinated (O(7)) water molecules generating 2D sheets along *bc* axis which are further cross linked to produce an interesting 3D supramolecular structure (Figure 8). In complex **4**, the coordination mode of H_5_L1 is identical to that present in **3**. H_5_L1 acts as pentadentate (N_2_O_3_) dianionic ligand (H_3_L^2−^), coordinating via two imine nitrogen atoms, a deprotonated phenoxide oxygen, a deprotonated alkoxide oxygen, and a protonated ethanolic OH group in the side arm of the ligand thereby bridging two copper(II) ions into dinuclear units. The second alkoxy group on either side of the Schiff-base ligand remains protonated and uncoordinated. As in compounds **1** and **3**, the two Cu(II) ions in each dinuclear unit are bridged via a phenoxide oxygen and a μ-1,1-N_3_ bridge.

The stereo-chemical environment at Cu(1) and Cu(2) can best be described as distorted square pyramidal (τ = 0.12), and distorted octahedral respectively. The coordination geometry in the basal plane of each copper(II) ion is defined by a phenoxide O-atom, (O(1)), an imine N-atom, (N(1)/N(2)), an alkoxide O-atom, (O(4)/O(3)), and azido nitrogen atoms (N(3)), with oxygen atom (O(5)/O(6)) of the ethanol OH group in the side arm of the Schiff base ligand/water at the axial position. There is a weak axial contact of Cu(2) with F(4) of BF_4_^−^, thus giving a distorted octahedral geometry at Cu(2). The sum of the angles in the basal plane of Cu(1) and Cu(2) are 359.97(18)° and 360.24(18)°, respectively, indicating planar arrangements at these metal centers for effective magnetic interaction.

In dinuclear units of **4**, the bridge angles at the phenoxide oxygen atom (O(1)) and the azido nitrogen atom (N(3)) are 100.44(17)° and 97.8(2)°, respectively. The sum of the angles at the oxygen atom of the phenoxide bridge, O(1), and the nitrogen atom of the azido bridge, N(3) are 358.54(8)° and 353.(3)°, respectively, indicating planar arrangements around O(1) and a slight distortion at N(3).

#### 2.2.5. [Cu_7_(H_3_L1^2−^)_2_(O)_2_(C_6_H_5_CO_2_)_6_]·6CH_3_OH·44H_2_O (**5**)

**Figure 9:**
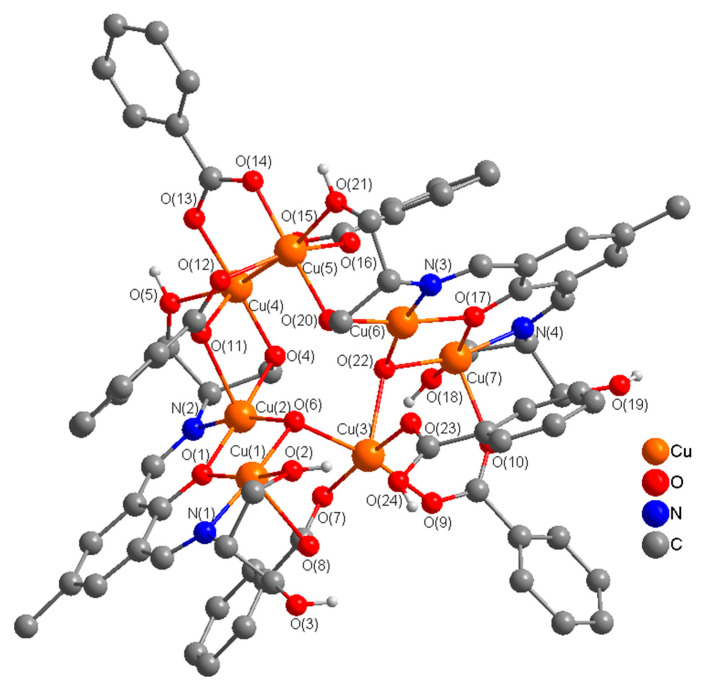
Perspective view of a heptanuclear complex [Cu_7_(H_3_L1^2−^)_2_(O)_2_(C_6_H_5_CO_2_)_6_] (**5)** with relevant numbering. H-atoms and solvent molecules are omitted for clarity.

There are fewer examples of heptanuclear copper(II) complexes in the literature in comparison with other nuclearities [23,109,110,111,112,113]. In complex **5,** two dinuclear [Cu_2_H_3_L1^2−^] units are connected to three copper(II) ions which are bonded to benzoate ions in a heptanuclear associated arrangement. In this complex, the benzoate ions exhibit four different types of bridging modes including (µ_4_-1,1,3,3-C_6_H_5_CO_2_), (µ_3_-1,1,3-C_6_H_5_CO_2_), (µ-1,3-C_6_H_5_CO_2_), and (µ-1,1-C_6_H_5_CO_2_), which is unprecedented. In our opinion, this constitutes the first report of a copper(II) complex in which benzoate ions exhibit four different types of bridging modes. In complex **5**, H_5_L1 acts as hexadentate (N_2_O_4_) dianionic ligand (H_3_L1^2−^) binding through two imine nitrogen atoms, a deprotonated phenoxide oxygen, and a deprotonated alkoxide oxygen, and two protonated ethanol (OH) groups in the side arms of the double Schiff base ligand, bridging two copper(II) ions into a dinuclear unit which is different from that in complexes **3** and **4** (pentadentate (N_2_O_3_)). One alkoxy group in one side arm of the Schiff-base ligand remains protonated and uncoordinated. In each dinuclear unit, two copper(II) ions are bridged through a phenoxide oxygen and μ_3_-O^2−^ bridges. Two dinuclear [Cu_2_] units are connected to three Cu^2+^ ions which are held in place by μ_3_-O^2−^, alkoxide O in the side arm of the ligand, and bridging benzoate ions that produce an interesting heptacopper structure. A perspective view of the **5** is presented in Figure 9. Important distances and angles are listed in Table S5.

The stereochemistry at Cu(1)/Cu(7) ion in each dinuclear unit can best be described as a distorted square pyramidal (τ (Cu(1) = 0.10 and Cu(7) = 0.04) with phenoxide O, imine N, alkoxide O, and an oxide O (μ_3_-O^2−^) in the equatorial plane and a benzoate O in the axial plane. The stereochemistry at Cu(2)/Cu(6) ion in each dinuclear can best be described as a distorted octahedral with phenoxide O, imine N, alkoxide O, and an oxide O (μ_3_-O^2−^) in the equatorial plane and benzoate O atoms in the axial plane. The stereochemistry at the other three copper centers, [Cu(3), (Cu(4), and Cu(5)] is also distorted octahedral. The sum of the angles in the basal plane of Cu(1), Cu(2), Cu(6), and Cu(7) are 359.7(2)°, 360.5(3)°, 360,6(2)°, and 356.6(2)° respectively, indicating almost planar arrangements around these metal centers. The sum of the angles at Cu(3), Cu(4), and Cu(5) which are held together by benzoate bridges are 358.4(2)°, 361.7(2)°, and 360,9(2)° respectively, indicating planar arrangements around these metal centers. In the dinuclear units, the bridge angles at the phenoxide oxygen (O(1)/O(17)) and μ_3_-O^2−^oxygen (O(6)/O(22)) are 99.7(2)° and 96.9(2)°/98.0(2)°, respectively. The sum of the angles around the phenoxide bridging O-atom, O(1)/O(17), and triply bridging μ_3_-O^2−^ O-atoms, (O(6)/O(22)) are 359.3(4)°/359.8(4)° and 315.8(2)°/317.4(2)° respectively, indicating planar arrangements at the phenoxide oxygen and a distorted pyramidal arrangement at the oxide oxygen atoms to allow effective magnetic exchange interaction between the Cu(1)/Cu(2) and Cu(6)/Cu(7) ions in the dinuclear units.

#### 2.2.6. [Cu_10_(H_3_L1^2−^)_4_(O)_2_(OH)_2_(C_6_H_5_CO_2_)_4_](C_6_H_5_CO_2_)_2_·20H_2_O (**6**)

**Figure 10:**
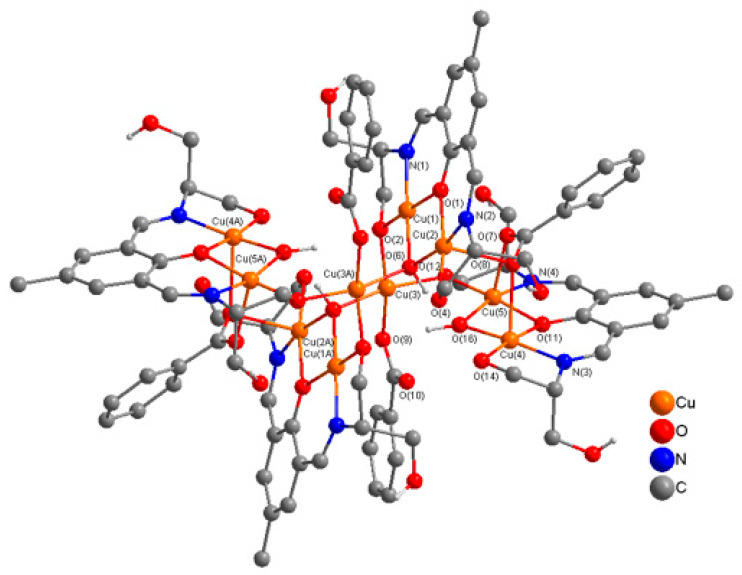
Perspective view of a cationic unit [Cu_10_(H_3_L1^2−^)_4_(O)_2_(OH)_2_(C_6_H_5_CO_2_)_4_]^2+^ in decanuclear complex **6** with relevant numbering. H-atoms, solvent molecules and uncoordinated [C_6_H_5_CO_2_^−^]_2_ anions are omitted for clarity.

In complex **6,** two cationic dinuclear [Cu_2_H_3_L1^2−^(OH)]^+^ units are linked to two cationic trinuclear units [Cu_3_H_3_L1^2−^(O^2−^)(C_6_H_5_CO_2_^−^)]^+^ via two alkoxide (µ-O-R^−^) ions in the side arms of the ligand and two triply bridging, µ_3_-1,1,3-C_6_H_5_CO_2_^−^ ions to generate a centrosymmetric decanuclear complex **6**. In **6**, four benzoate ions (µ_3_-1,1,3-C_6_H_5_CO_2_^−^) act as intra-dimer and inter-dimer bridges, generating a decacopper complex. In complex **6**, H_5_L1 acts as pentadentate (N_2_O_3_) dianionic ligand (H_3_L1^2−^) as in **3** and **4**, binding through two imine nitrogen atoms, a deprotonated phenoxide oxygen, and a deprotonated alkoxide oxygen, and a protonated ethanol (OH) group in the side arm of the double Schiff base ligand. This is different from that in **5,** where H_5_L1 acts as a hexadentate (N_2_O_4_) dianionic ligand. Here one ethanol group (OH) in the side arm of the ligand is deprotonated and acts as a bridge between dinuclear and trinuclear units to produce a decanuclear complex. One ethanol group (OH) of the side arm remains protonated and coordinates to the Cu(II) ion in a dinuclear unit. Two ethanol groups of the side arms of the ligand remain protonated and uncoordinated. Two copper(II) ions in a dinuclear unit are bridged by a phenoxy oxygen and a hydroxy oxygen (µ-OH^−^), whereas two Cu(II) ions in another dinuclear unit are bridged by a phenoxide oxygen and a µ_3_-O^2−^ ion, which acts as an intradinuclear bridge as well as a link between a dinuclear unit and a trinuclear unit forming the decanuclear supramolecular architecture **6**. A perspective view of the **6** is presented in Figure 10 and important distances and angles are listed in Table S6.

**Figure 11:**
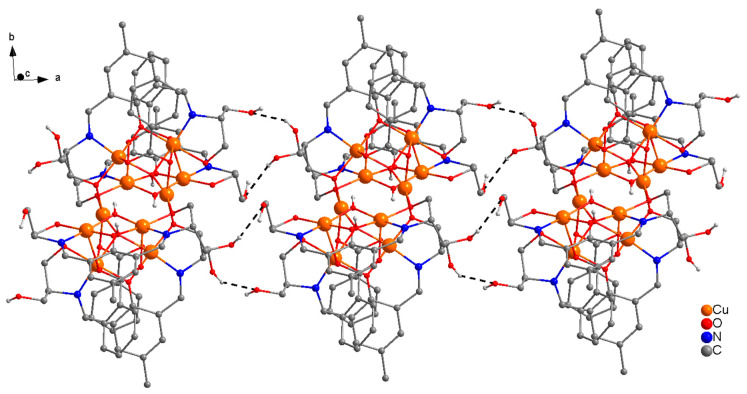
Perspective view of a portion of 1D-single chain along *a*-axis in the structure of **6**.

H-bonding forces, which are so important for stabilizing the structure of proteins and other biomolecules in living systems, play a prominent role in the stability of **6** and in the formation of 1D-single chains. There are remarkably strong intramolecular H-Bonding interactions [O(4)---O(14) = 2.477 Å, O(6)---O(16) = 2.623 Å, O(10)---(O(16) = 2.787 Å] between µ-OH(O(16)), µ_3_-O^2^ (O(6)), µ_3_-1,1,3-C_6_H_5_CO_2_^−^ (O(10)),and OH(O(4) and O(14)) in the side arms of the ligand, which stabilize the structure. Complex **6** grows along the *a*-axis, generating an unprecedented supramolecular 1D-single chain through a network of H-bonds [O(3)---O(5) = 2.797 Å and O(13)---O(15) = 2.759 Å) between four protonated uncoordinated OH groups (O(3), O(5), O(13), O(15)) in the side chains of the ligands (see Figure 11).

The stereochemistry at Cu(1), Cu(4), and Cu(5) can best be described as square pyramidal (τ (Cu(1) = 0.04, Cu(4) = 0.10, and Cu(5) = 0.06) with phenoxide O, imine N, alkoxide O, and an oxide O (μ_3_-O^2−^)/hydroxy (µ-OH) in the equatorial plane and a benzoate O in the axial plane. The stereochemistry at the Cu(3) ion can best be described as a distorted square planar (τ = 0.05) with one benzoate O, two alkoxide O, and one hydroxide O (μ-OH^−^) in the equatorial plane. The geometry at Cu(2) is distorted octahedral with phenoxide O, imine N, alkoxide O, and an oxide O (μ_3_-O^2−^) in the equatorial plane and benzoate O atoms in the axial plane. The sum of the angles in the basal plane of Cu(1) to Cu(5) are 360.2(4)°, 358.3(3)°, 364,1(3)°, 359.5(4)° and 358.7(4)° respectively, indicating almost planar arrangements around these metal centers. In the dinuclear units, the bridge angles at the phenoxide oxygen (O(1)/O(11)) are 97.5(4)°/97.3(4)°, similar to **5**. The Cu(4)-O(16)-Cu(5) bridge angle at doubly bridged hydroxide (µ-OH) O is 97.3(4)° and Cu(1)-O(6)-Cu(2), Cu(2)-O(6)-Cu(3A), Cu(1)-O(6)-Cu(3A) bridge angles at the triply bridged oxide (µ_3_-O^2−^) O are 97.1(4)°, 102.0(5)°, and 118.9(4)° respectively. The sum of the angles around the phenoxide bridging O-atoms, O(1) and O(11) are 359.1(8)°and 353.3(8)° respectively, indicating planar and a distorted planar arrangements at these atoms. The sum of the angles at alkoxide O-atoms, O(2) and O(12) are 345.9(7)° and 346.9(7)° indicating a distorted pyramidal arrangement at oxide oxygen atoms.

### 2.3. Magnetic Properties

**Figure 12:**
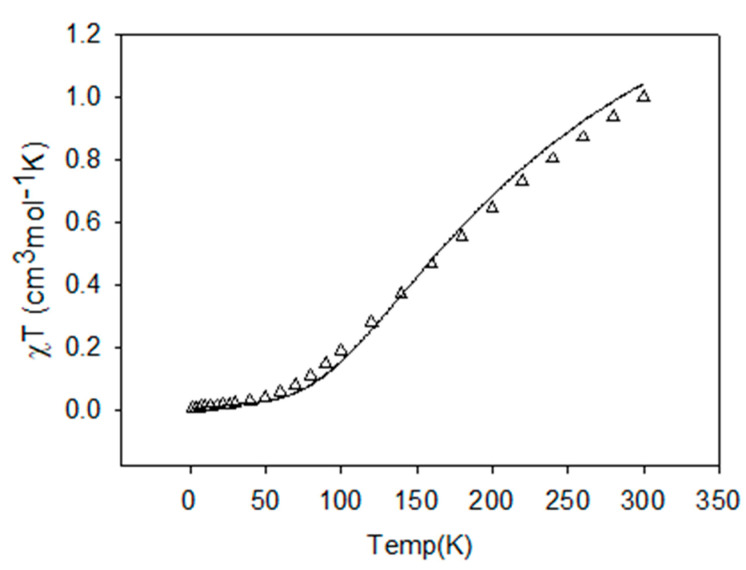
Plots of *χ_M_T* (triangles) vs. *T* per mole of [Cu_2_] unit for **1**. The solid line is the best fit to the experimental data.

**Figure 13:**
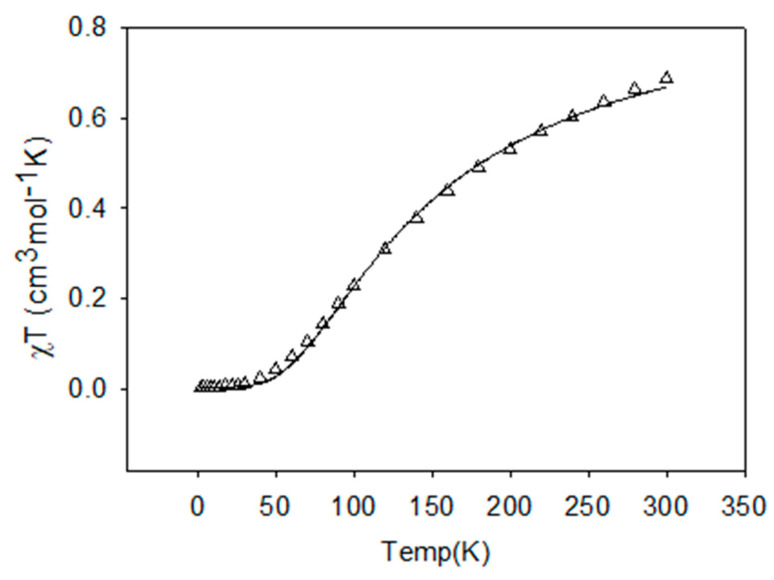
Plots of *χ_M_T* (triangles) vs. *T* per mole of [Cu_2_] unit for **3**. The solid line is the best fit to the experimental data.

**Figure 14:**
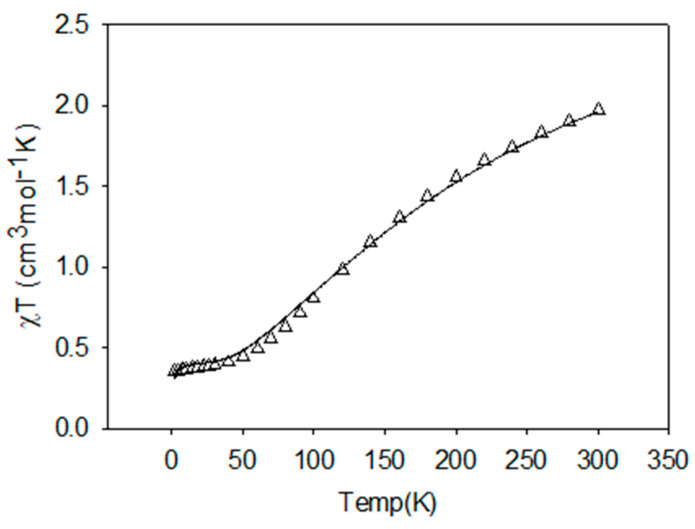
Plots of *χ_M_T* (triangles) vs. *T* per mole of [Cu_2_] unit for **5**. The solid line is the best fit to the experimental data.

In the compounds under investigation, the Cu-Cu distances are quite short. The copper ions in the dinuclear units are bridged via single atom-phenoxy oxygen atom of the ligand and end-on azido µ-1,1-N_3_/hydroxy (µ-OH^−^)/oxide (µ-O^2−^) bridges and are likely to result in magnetic exchange interactions between closely placed metal centers. Variable temperature magnetic studies have been carried out on **1**, **3**, and **5,** and the results of our investigations are presented as the best fit curves along with experimental data in the Figure 12, Figure 13 and Figure 14 respectively. Based on the structural information, we anticipate the presence of strong antiferromagnetic spin exchange interactions via the PhO^−^ and N_3_^−^/hydroxy (µ-OH^−^)/oxide (µ-O^2−^) bridges within the [Cu_2_] units in these complexes, which involve all equatorial positions of the metals and bridging units.

Despite the butterfly, cubane type structure of complex **1**, the basic arrangement is comprised of two almost planar dinuclear fragments joined axially through long (2.4 Å) and a very long (3.02 Å) axial contacts. Theoretically, these axial contacts are orthogonal and so contribute little to overall antiferromagnetic exchange [114]. Looking at the Cu–O–Cu and Cu–N–Cu angles, one would expect net antiferromagnetic (AF) exchange, as is observed experimentally. The best fit to a dinuclear model is not brilliant, and gives g = 2.03, J = −278 cm^−1^, temperature independent magnetism (TIP) = 445 × 10^−6^ cm^3^ mol^−1^, and fraction paramagnetic impurity (ρ) = 0.003, 10^2^ R = 9.38. (R represents the agreement factor of data fitting which can be defined as R = ∑[(χ_M_T)_exp_ − (χ_M_T)_calc_]^2^/∑[(χ_M_T)_exp_]^2^). This was the best fit possible, based on the projected exchange model.

H_ex_ = −J{S_1_·S_2_}

Using Hatfield’s correlation for Cu–O–Cu angle versus exchange [115], J_calc_ = −332 cm^−1^, it is possible that the azide is responsible for a small ferromagnetic contribution, which would agree with our azide correlation (*vide supra)* [116,117].

Complex **3** contains a simple dinuclear unit with two in plane active bridges, both connecting the d_x_^2^-_y_^2^ metal magnetic orbitals. The fit is good, indicating overall AF coupling, and gives g = 2.13(1), J = −177.3(2) cm^−1,^ TIP = 100 × 10^−6^ cm^3^ mol^−1^, ρ = 0.004, 10^2^R = 2.41 (Agreement factor R is defined as, R = [Σ(ᵡ_obs_ − ᵡ_calc_)^2^/Σᵡ_obs_^2^]^1/2^).

H_ex_ = −J{S_1_·S_2_}

The Cu–O–Cu angle (100.9°) suggests AF exchange, while the Cu–N_3_–Cu angle (100.1°) is just in the Ferromagnetic realm. Since Ferromagnetic (F) and antiferromagnetic (AF) are additive and of opposite sign, one would expect the AF J value to be less than predicted based on Hatfield’s correlation. For the Cu–O–Cu angle J_calc_ = ~−250 cm^−1^, in complete agreement [115].

Complex **5** breaks down nicely into two isolated parts both expected to be AF. The fit assumes that all J values are the same, which is not unreasonable given the bridges and the Cu–O–Cu angles. The benzoates are not influencing exchange in any significant way.

H_ex_ = −J{S_1_·S_2_ + S_2_·S_3_ + S_1_·S_3_ + S_2_·S_4_ +S_5_·S_6_ +S_6_·S_7_}

The fit is good, giving g = 2.09(2), J_av_ = −204(7) cm^−1^, TIP = 340 × 10^−1^ cm^3^ mol^−1^, θ = −0.6 K, 10^2^R = 2.96.

**Figure 15:**
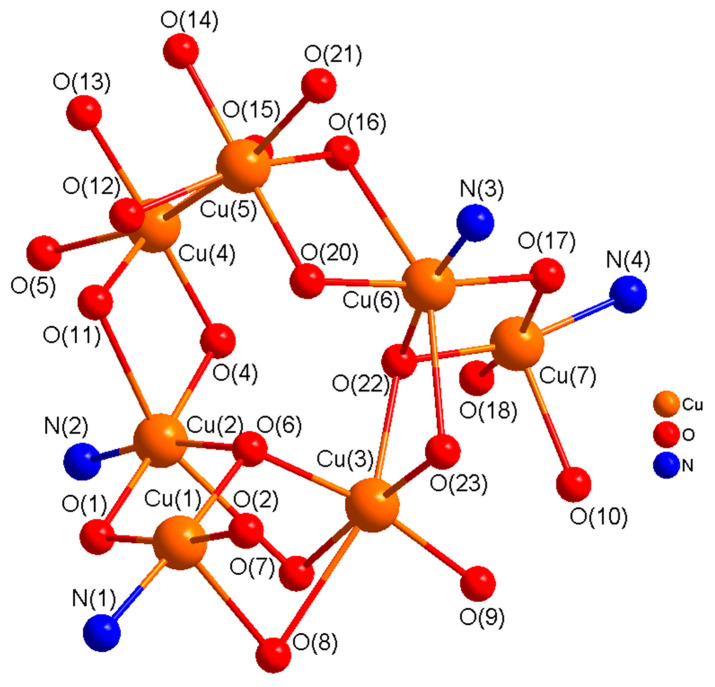
The structure of **5** showing only the copper atoms and the donor atoms.

For clarity, the structure of **5** showing only the metal centers and the coordinating atoms is shown in Figure 15.

### 2.4. Magneto-Structural Relationships

**Figure 16:**
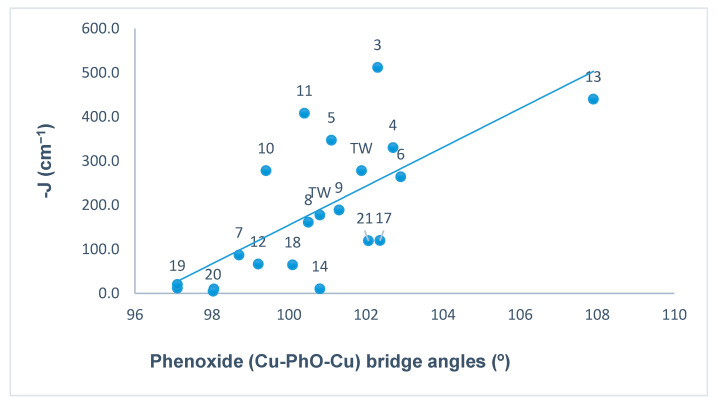
Plot of the antiferromagnetic interaction (−J) vs. the Cu–OPh–Cu angle in dinuclear. (μ-phenolate/μ-azido bridged) copper(II) complexes.

**Figure 17:**
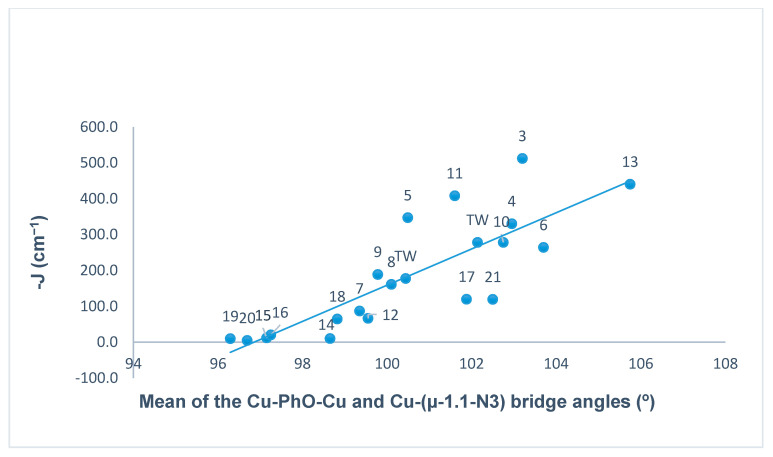
Plot of the antiferromagnetic interaction (–J) vs. the averaged Cu–OPh–Cu and Cu–(μ-N_3_) angle in dinuclear (μ-phenolate/μ-azido bridged) copper(II) complexes.

**Figure 18:**
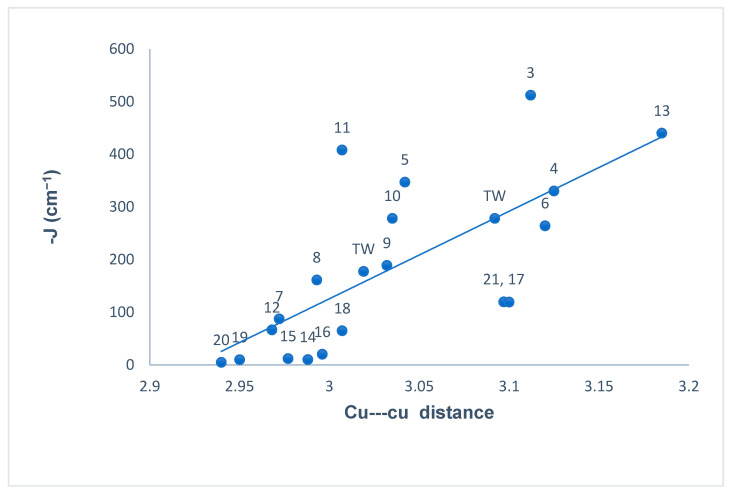
Plot of the antiferromagnetic interaction (−J) vs. the Cu---Cu distance (d) in dinuclear (μ-phenolate/μ-azido bridged) copper(II) complexes.

In doubly bridged [Cu_2_(µ-OPh)(µ-1,1-N_3_)] copper(II) complexes, the nature (ferromagnetic/ antiferromagnetic) and the magnitude of the magnetic spin exchange interaction (J) depends primarily on the bridge angles, but other important factors such as the intermetallic distance (d), bond distance in the equatorial plane, stereochemistry, and distortion from planarity in the mean plane of dinuclear core can also influence the magnitude of the coupling constant (J) [118]. In order to illustrate the magneto-structural trends, we have compiled the magnetic data of all the copper(II) complexes (Table 1) from the literature that contain endogenous phenoxide bridge and exogenous EO µ-azido bridge along with two new compounds (**1** and **3**) reported in this study. The relationships between the antiferromagnetic coupling constant (−J) and phenoxide bridge angle (Cu–PhO–Cu), average bridge angles of µ-phenoxide bridges and µ-azido bridges, and the Cu–Cu distance (d) are represented in Figure 16, Figure 17 and Figure 18 respectively, and summarized in Table 1.

H_3_L and H_5_L1 are the ligands used in this publication. H_3_L2, 2,6-bis[[(2-hydroxyethyl) imino] methyl]-4-methylphenol. L3, Schiff base of 2-hydroxy-5-methylisophthalaldehyde and dimethylamino-1-propylamine. L4 (Fdmen), Schiff base of 2,6-diformyl-4-methylphenol with 1,1-dimethylethylenediamine. L5 (Fmap), 2,6-bis(*N*-(2-pyridylmethyl) formimidoyl)-4-methylphenolate. L6, Schiff-base of 4-methyl-2,6-diformylphenol and the 1,2-diaminoethane. L7, 4-methyl-2,6-bis[*N*-(2-pyridylethyl)formimidoyl]phenolate. L8, 4-Methyl-2,6-bis[*N*-(2-methylthioethyl) formimidoyl]phenolate. L9, 2,6-diformyl-4-methyl phenol-di(benzoylhydrazone). L10, 2,6-bis(dipyridylmino)phenol. L11, 2-[1-(2-dimethylamino-ethylimino)-ethyl]-phenol. L12, 2-[1-(2-diethylamino-ethylimino)-ethyl]-phenol. L13 = 2,6-bis[*N*-(2-pyridylethyl)formidoyl]-4-ethylphenol. L14, *N*,*N*-bis(3,5-dimethyl-2-hydroxybenzyl)-*N*′,*N*′-dimethyl-1,3-diaminopropane. L15, *N*,*N*-bis(3,5-dimethyl-2-hydroxybenzyl)-*N*′,*N*′-dimethyl-1,2-diaminoethane. L16, *N*,*N*-bis(3,5-dimethyl-2-hydroxybenzyl)-*N*′,*N*′-diethyl-1,2-diaminoethane.L17, 2,6-bis[{[(2-hy-droxybenzyl)(*N*,*N*-(dimethylamino)ethyl]amino} methyl]-4-methylphenol.

For the two complexes (**1** and **3, i**n Table 1, **1** and **2**) reported in this study, a strong antiferromagnetic interaction (−J = 278, 177.3 cm^−1^ respectively) occurs within the dinuclear [Cu_2_(µ-OPh)(µ-1,1-N_3_)] core where the Cu-N and Cu-O distances in the equatorial plane fall in the ranges of 1.915–1.980 Å and 1.9341–1.998 Å respectively. These are quite short and are within the plane of the magnetic orbitals of both metals (dx^2^-y^2^) which are approximately parallel, and are responsible for effective coupling between copper(II) centers in each dinuclear unit. Based on the bridge angles, it is anticipated that the bridging moieties (µ-phenolato and µ-azido) provide counter complementary contributions to the magnetic exchange interaction between the copper(II) centers in [Cu_2_(OPh)(µ-1,1-N_3_)] core [127,128].

The phenoxide bridge angle of 101.88° (**1**)/100.80° (**3**) and the µ-azido bridge angles of 102.4° (**1**)/100.07° (**3**) in these complexes are expected to mediate antiferromagnetic and ferromagnetic spin coupling respectively between the copper centers, based on previous studies [111,118]. This is consistent with the reported data for µ-phenolato/µ-azido bridged copper(II) complexes presented in Figure 16 and Figure 17. It is a well- established fact that for bis(µ-phenolato) bridged copper(II) complexes if the bridge angle is less than the critical angle of ~97°/98°, the spin coupling constant (J) between the copper centers is dominantly ferromagnetic, and for larger angles an antiferromagnetic interaction is expected [36,129]. For dinuclear complexes involving a µ-azido bridge, it has been established that the nature of the spin coupling constant (J) is dependent on the Cu–(µ-N_3_)–Cu bridge angle, and that the magnitude of ferromagnetic coupling (J_ferro_) decreases with increasing bridge angle (critical angle 104º (according to theoretical calculation) [130] or 108° (experimental studies) [115,116]).

The antiferromagnetic spin coupling constant (−J) plotted against the phenolate bridge angle and the averaged bridge angle of phenolate and azido for all the reported copper(II) complexes is shown in Figure 16 and Figure 17 respectively. Figure 16 shows a relationship between −J and phenolate bridge angles with reasonable linear character. A graph of −J vs. averaged bond angles (Figure 17) shows a much better linear trend between coupling constant −J and averaged bridge angles for majority of the complexes including two in the present study, with only a few exceptions. The complexes (**1** and **3**) reported in this study lie close to the line of best fit. In these double bridged (phenoxide and end-on azido) copper(II) complexes, the averaged bridge angles lies in the range 96.29–105.75° and the coupling constant (−J) lies in the range, J = −4.54 cm^−1^ for small angle (96.69°) to J = −440 cm^−1^ for the large angle (105.75°) with the exception of compound **3** in Table 1 where the average bridge angle is 103.20° and J = −512 cm^−1^. While these plots show realistic trends, with dominant linear character, it is necessary to stress that the J values are based on the sum of two counter-complementary exchange contributions, where the individual bridges have linear variations with angle, which are different. This helps to explain why the general appearance of the averaged data plotted in Figure 17 look more linear than those in Figure 16.

Figure 18 summarizes the trend in exchange integral as a function of Cu–Cu distance listed in Table 1. A reasonably linear relationship is evident for majority of the complexes. This agrees with the expected increase in both bridge angles, resulting in an increase of the antiferromagnetic contribution as reported previously [36,41,115,116,129,130].

### 2.5. Powder X-ray Diffraction Studies

In an attempt to characterize the bulk powder, the XRD patterns of **1**, **3** and **5** were collected. (Figures S3–S5). The XRD patterns were collected and compared to the calculated pattern generated from the single-crystal X-ray structure [131]. For **3** and **5**, the form of the diffraction curve for the observed pattern was similar to that of the calculated pattern. There were minor differences (i.e., intensity variations, changes in peak full-width, and peak position) between the calculated and observed peaks. Peak shifts are an artifact, given that the powder data were collected at room temperature, while the calculated pattern was based on structural data from −100 °C. This difference may change the unit cell dimensions and shift peak positions along the 2θ axis. For **1**, peak differences may result from the sample being grinded prior to characterizing. Mechanical grinding could alter the crystallite structure, possibly through the loss of solvent in the lattice. Although the XRD powder pattern shows consistency to that of the calculated powder pattern, the measurement also does not reveal amorphous content that may be present within the sample. Hence, the XRD powder data are not useful for commenting on the purity of the crystalline phase(s) present.

## 3. Materials and Methods 

### 3.1. Physical Measurements

Infrared spectra were recorded as Nujol mulls using a Perkin Elmer FT-IR instrument, and Uv/Vis spectra of the powdered compounds were obtained as Nujol mulls or in solution using a Cary 5E spectrometer. Micro-analyses were carried out using a Leco CHNS-Analyzer. Variable temperature magnetic data (2–300 K) were obtained using a Quantum Design MPMS5S SQUID magnetometer with a field strength 0.1 T. Background corrections for the sample holder assembly and diamagnetic components of the complexes were applied. X-ray powder patterns were collected using a Rigaku Miniflex 600 X-ray Diffractometer. The radiation used was Cu Kα radiation (λ = 1.54059 Å).

### 3.2. Material

First, 2,6-Diformyl-4-methylphenol (**DFMP**) was isolated using the reported method [132]; 1-amino-2-propanol (**AP**), and 2-amino-1,3-propanediol (**APD**) were supplied by Aldrich. All other chemicals used (solvents and metal salts) were analytical or reagent grade and were employed without further purification. Schiff base ligands were prepared in situ by metal catalyzed self-assembly. 

### 3.3. Synthesis of the Coordination Complexes

Caution: Azide and perchlorate complexes of metal ions involving organic ligands are potentially explosive. Only small quantities of the complexes should be prepared, and these should be handled with care.

In some cases, there is a difference between the most reasonable formula based on the elemental analysis (analytical formula) and that obtained from X-ray crystallography. In these compounds the CHN analysis showed a different number of solvent molecules (methanol and water) compared with the X-ray formulae, as the analysis was carried out on air dried samples due to their potential explosive nature. For consistency, the X-ray formulae will be used in the discussion. For compound **1,** the X-ray formula is [Cu_4_(HL^2−^)_2_(N_3_)_4_]∙4CH_3_OH∙56H_2_O and the analysis formula is [Cu_4_(HL^2−^)_2_(N_3_)_4_]∙6H_2_O. For compound **2,** the X-ray formula is [Cu_4_(HL^2−^)_2_(O)_2_(H_2_O)_2_] and the analysis formula is [Cu_4_(HL^2−^)_2_(O)_2_(H_2_O)_2_]·12H_2_O. For compound **3,** the X-ray formula is [Cu_2_(H_3_L1^2−^)(H_2_O)(NO_3_)(N_3_)] and the analysis formula is [Cu_2_(H_3_L1^2−^)(H_2_O)(NO_3_)(N_3_)]·CH_3_OH·0.8H_2_O. For compound **5,** the X-ray formula is [Cu_7_(H_3_L1^2−^)_2_(_-_O)_2_(C_6_H_5_CO_2_)_6_]·6CH_3_OH·44H_2_O and the analysis formula is [Cu_7_(H_3_L1^2−^)_2_(O)_2_(C_6_H_5_CO_2_)_6_]·10H_2_O. For compound **6,** the X-ray formula is [Cu_10_(H_3_L1^2−^)_4_(O)_2_(OH)_2_(C_6_H_5_CO_2_)_4_](C_6_H_5_CO_2_)_2_**·**20H_2_O and the analysis formula is [Cu_10_(H_3_L1^2−^)_4_(O)_2_(OH)_2_(C_6_H_5_CO_2_)_4_](C_6_H_5_CO_2_)_2_**·**13H_2_O.

#### 3.3.1. [Cu_4_(HL^2−^)_2_(N_3_)_4_]·CH_3_OH·14H_2_O (**1**)

First, 1-Amino-2-propanol (**AP**) (0.08 g, 1.0 mmol) dissolved in 3 mL of methanol was added dropwise to a solution of 2,6-diformyl-4-methylphenol (**DFMP**, 0.09 g, 0.50 mmol) in hot methanol (10 mL) while stirring under reflux. The yellow solution formed was refluxed for 30 min and a solution of Cu(BF_4_)_2_·6H_2_O (1.0 mmol, 0.35 g) in methanol (5 mL) was added dropwise. The reaction mixture (green) was refluxed for 10 min, and a solution of NaN_3_ (0.07 g, 1.0 mmol) in a hot methanol (10 mL) was added dropwise. The color of the reaction mixture changed to dark green and it was refluxed further for 2.0 h. The green solution was filtered hot, and the filtrate was kept unperturbed at room temperature for slow evaporation. After two weeks, dark green crystals suitable for X-ray studies were obtained and some were kept in the mother liquor for X-ray analysis. The bulk sample was separated from the mother liquor and washed with methanol (2 × 2 mL) and air dried at ambient temperature. IR spectrum: 3423 cm^−1^ (υ(OH) H_2_O and CH_3_OH), 2093, 2037 cm^−1^ (υas (N_3_)), 1637 cm^−1^ (υ(C=N)). UV-Vis Spectrum: 330 nm (s), 370 nm (sh) (Cu-azide and Cu-ligand charge transfer transitions respectively), and 625 nm (d-d transition). Yield: 0.14 g, 48%, based on **DFMP**. Elemental analysis (air dried sample): Found (%): C, 32.95; H, 4.97; N, 21.14. Calcd (%) for [Cu_4_(C_15_H_20_N_2_O_3_)_2_(N_3_)_4_]∙6H_2_O, C, 33.33; H, 4.85; N, 20.74. 

#### 3.3.2. [Cu_4_(L^3−^)_2_(OH)_2_(H_2_O)_2_]·CH_3_OH·11H_2_O (**2**)

Complex **2** was prepared by exactly the same method as used for **1** by replacing Cu(BF_4_)_2_·6H_2_O with CuCl_2_·6H_2_O and adding 10 drops of triethylamine in the absence of NaN_3_. Dark green crystals suitable for x-analysis were obtained by keeping the reaction mixture unperturbed at ambient temperature for 5 weeks. IR spectrum: 3450 cm^−1^, 3341 cm^−1^ (υ(OH) H_2_O and CH_3_OH), 1652 cm^−1^, 1637 cm^−1^ (υ(C=N)). UV-Vis Spectrum: 375 nm (Cu-ligand charge transfer transitions) and 735 nm (d-d transition). Yield: 0.085 g, 40%, based on **DFMP**. Elemental analysis (air dried sample): Found (%): C,32.75; H, 5.86; N, 5.48. Calcd (%) for [Cu_4_(C_15_H_19_N_2_O_3_)_2_((OH)_2_(H_2_O)_2_]·12H_2_O: C, 33.02; H, 6.28; N, 5.14. 

#### 3.3.3. [Cu_2_(H_3_L1^2−^)(N_3_)(H_2_O)(NO_3_)] (**3**)

First, 2,6-Diformyl-4-methylphenol (**DFMP**, 0.09 g, 0.50 mmol) dissolved in hot methanol (10 mL) was added to a solution of 2-amino-1,3-propanediol (**APD**) (0.09 g, 1.0 mmol) in the same solvent (5 mL). The yellow solution of the Schiff-base ligand (H_5_L1) formed was stirred under reflux for 30 min, and a solution of Cu(NO_3_)_2_·3H_2_O (0.24 g, 1.0 mmol) in methanol (5 mL) was added to it dropwise. The solution changed from brown to green in about 5 min. The resulting green solution was refluxed for 10 min and a solution of NaN_3_ (0.070 g, 1.0 mmol) in hot methanol (10 mL) was added dropwise. The color of the reaction mixture changed to dark green and was refluxed further for 1.5 h. A clear green solution was filtered hot and the filtrate was left undisturbed at ambient temperature for slow evaporation. After four weeks, dark green crystals suitable for x-ray analysis were formed, separated from the mother liquor, and washed with methanol (2 × 2 mL). IR spectrum: 3392, 3322 cm^−1^ (υ(OH) H_2_O), 2110, 2074, 2050 cm^−1^ (υas (N_3_)), 1648, 1634 cm^−1^ (υ(C=N)). UV-Vis Spectrum: 378 nm (s), and 410 nm (sh) (Cu-azide and Cu-ligand charge transfer transitions respectively), and 625 nm (d-d transition). Yield: 0.16 g, 54%, based on **DFMP**. Elemental analysis (air dried sample): Found (%): C, 31.73; H, 3.97; N, 13.46. Calcd (%) for [Cu_2_(C_15_H_19_N_2_O_5_)(H_2_O)(NO_3_)(N_3_)]·CH_3_OH·0.8H_2_O: C, 31.87; H, 4.45; N, 13.94. 

#### 3.3.4. {[Cu_2_(H_3_L1^2−^)(H_2_O)(BF_4_)(N_3_)]·H_2_O}_n_ (**4**)

Compound **4** was obtained in a similar manner as compound **3**. In this case after adding the NaN_3_ solution to the reaction mixture of **DFMP** (0.50 mmol), 2-amino-1,3-propanediol (1.0 mmol), and Cu(BF_4_)_2_·6H_2_O (0.35 g, 1.0 mmol), the mixture was further refluxed for 2.0 h and left at room temperature undisturbed for slow evaporation. After one week, very nice crystals suitable for X-ray studies separated from the dark green solution. The crystals used for X-ray studies were kept in the mother liquor. The remaining crystals were separated and washed with methanol (2 × 2 mL). IR spectrum: 3361 cm^−1^ (υ(OH) H_2_O), 2114 cm^−1^, 2080 cm^−1^ (υas (N_3_)), 1646 cm^−1^, 1635 cm^−1^ (υ(C=N)). UV-Vis Spectrum: 327 nm, and 370 nm (Cu-azide and Cu-ligand charge transfer transitions respectively), and 625 nm (d-d transition). (Yield: 0.18 g, 54%). Elemental analysis: Found (%): C, 30.50; H, 4.11; N, 11.44. Calcd (%) for {[Cu_2_(C_15_H_19_N_2_O_5_)(H_2_O)(BF_4_)(N_3_)]·H_2_O}_n_: C, 30.01; H, 3.87; N, 11.69. 

#### 3.3.5. [Cu_7_(H_3_L1^2−^)_2_(O)_2_(C_6_H_5_CO_2_)_6_]·6CH_3_OH·44H_2_O (**5**)

First, 2,6-Diformyl-4-methylphenol (0.17 g, 1.0 mmol) dissolved in hot methanol (15 mL) was added to a solution of 2-amino-1,3-propanediol (**APD**) (0.18 g, 2.0 mmol) in methanol (10 mL). The yellow solution of the Schiff-base ligand (H_5_L1) was then refluxed for 30 min and Cu(ClO_4_)_2_·6H_2_O (0.92 g, 0.25 mmol) dissolved in hot methanol (10 mL) was added to it dropwise with stirring under reflux. The bright green solution formed was refluxed further for 10 min and a solution of sodium benzoate (C_6_H_5_CO_2_Na) (0.30 g, 2.0 mmol) in hot methanol (15 mL) was added dropwise. After refluxing the reaction mixture for 10 min, a solution of triethylamine (0.20 g, 2.0 mmol) dissolved in 5 mL of methanol was added dropwise, which caused a color change of the reaction mixture to brownish green. It was stirred under reflux for 2.0 h and filtered hot. The filtrate was left unperturbed at ambient temperature for slow evaporation. After three weeks some colorless crystals, which were possibly of sodium benzoate, separated and were filtered off. 5 mL of ethanol and 2 mL of water was added to the filtrate and left at room temperature for slow evaporation. After two weeks green crystals suitable for X-ray analysis formed and were kept in the mother liquor. The crystals of the bulk sample were separated from the mother liquor and washed with methanol (2 × 2 mL). IR spectrum: 3400 cm^−1^ (υ(OH) H_2_O and CH_3_OH), 1645, 1608 cm^−1^ (υ(C=N)). UV-Vis Spectrum: 330 nm and 370 nm (Cu-azide and Cu-ligand charge transfer transitions respectively), and 630 nm (d-d transition). Yield: 0.45 g, 43 % based on **DFMP**. Elemental analysis on the bulk air-dried sample: Found (%): C, 42.77; H, 4.03; N, 2.54. Calcd (%) for [Cu_7_(C_15_H_20_N_2_O_5_)_2_(O)_2_(C_6_H_5_CO_2_)_6_]·10H_2_O: C, 43.23; H, 4.53; N, 2.80. 

#### 3.3.6. [Cu_10_(H_3_L1^2−^)_4_(O)_2_(OH)_2_(C_6_H_5_CO_2_)_4_](C_6_H_5_CO_2_H)_2_·20H_2_O (**6**)

Complex **6** was prepared by using the same method as used for **5** by reacting Cu(NO_3_)_2_∙3H_2_O (0.58 g, 3 mmol) with the Schiff base prepared by reacting 2,6-diformyl-4-methyphenol (0.17 g, 1 mmol) and 2-amino-1,3-propanediol (0.18 g, 2.0 mmol), C_6_H_5_CO_2_Na (0.30 g, 2.0 mmol), and triethylamine (0.22 g, 2.2 mmol). IR spectrum: 3289 cm^−1^ (υ(OH) H_2_O), 1644, 1628 cm^−1^ (υ(C=N)). UV-Vis Spectrum: 320 nm (Cu-ligand charge transfer transitions respectively), and 638 nm (d-d transition). Yield: 0.26 g, 35%, based on **DFMP**. Found (%): C, 42.01; H, 4.93; N, 4.20. Calcd. for [Cu_10_(H_3_L1)_4_(O)_2_(OH)_2_(C_6_H_5_CO_2_)_4_](C_6_H_5_CO_2_)_2_**·**13H_2_O: C, 42.31; H, 4.80; N, 3.87.3.

### 3.4. X-ray Crystallography

Suitable single crystals for X-ray diffraction studies were obtained for **1–6**. Crystal data for the compounds were collected by the same method by mounting a crystal onto a thin glass fiber from a pool of Fluorolube^TM^ and immediately placing it under a liquid N_2_ cooled stream on a Bruker AXS diffractometer upgraded with an APEX II CCD detector. The radiation used was graphite monochromatized Mo Kα radiation (λ = 0.7107 Å). The lattice parameters were optimized from a least-squares calculation on carefully centered reflections. Lattice determination, data collection, structure refinement, scaling, and data reduction were carried out using APEX3 Version 2018.11 software package [133,134]. The data were corrected for absorption using the SCALE program within the APEX3 software package [133,134]. The structures were solved using SHELXT [135]. This procedure yielded a number of the C, N, Cu, O, F and B atoms. Subsequent Fourier synthesis yielded the remaining atom positions. The hydrogen atoms were fixed in positions of ideal geometry (riding model) and refined within the XSHELL software package [136]. The final refinement of each compound with anisotropic thermal parameters on all nonhydrogen atoms was performed using OLEX2-1.2 [137]. The crystal data for compounds **1**–**6** are given in Table 2. Crystallographic data for the structures has been deposited with the Cambridge Crystallographic Data Centre as supplementary publication nos: CCDC 1944278-1944283. Copies of the data can be obtained, free of charge on application to CCDC, 12 Union Road, Cambridge CB2 1EZ, UK (fax, +44-(0)1223-336033; or e-mail, deposit@ccdc.cam.ac.uk).

## 4. Conclusions

We reported the coordination versality of two double Schiff base ligands H_3_L (potentially pentadentate (N_2_O_3_) tri-anionic) and H_5_L1 (potentially heptadentate (N_2_O_5_) penta-anionic) with a high degree of conformational flexibility, having one or two ethanoate hydroxy groups in the side arms and a potential to coordinate in a convergent and a divergent fashion with Cu(II) ions. Based on the reaction conditions, the nature of the anion, and the stereochemical requirements of the metal, these ligands were shown to exhibit diverse coordination versatility. In complex **1**, H_3_L acts as tetradentate (N_2_O_2_) dianionic ligand (HL^2−^), whereas in **2,** it acts as a pentadentate (N_2_O_3_) tri-anionic (L^3−^) ligand, holding two Cu(II) ions in close proximity for magnetic exchange interaction. Reactions of copper(II) ions with H_5_L1 under varied conditions resulted in the formation of dinuclear (**3**), polynuclear (**4**), heptanuclear (**5**), and decanuclear (**6**) complexes, depending upon the anions. This clearly demonstrated the significant effect of the nature of an anion on the nuclearity of the complex produced. In dinuclear complex **3** and polynuclear complex **4**, which grew into very fascinating 3D-network structures through H-bonding, and in decanuclear complex **6**, H_5_L1 acted as a pentadentate (N_2_O_3_) dianionic dinculeating ligand (H_3_L1^2−^). In complex **6**, ethanolic (OH) groups in the side arms of the ligand remained protonated and uncoordinated, and were involved in symmetrical H-bonding, generating 1D-single chains of decanuclear cationic [Cu_10_(H_3_L1^2−^)_4_(μ_3_-O)_2_(μ-OH)_2_(μ_3_-1,1,3-C_6_H_5_CO_2_)_4_]^2+^, units. In heptanuclear complex **5**, H_5_L1 behaved as a hexadentate (N_2_O_4_) dianionic ligand (H_3_L1^2−^). In complex **5**, benzoate ions exhibited four different types of coordination modes which, in our opinion, constitutes a novel discovery. The magnetic coupling in complexes **1**, **3**, and **5** was dominated by the strong antiferromagnetic interaction mediated by the phenoxide bridge within the [Cu_2_] moieties with coupling constants ranging from −177 to −278 cm^−1^ (in the *H =* −*J*(*S*_1_*S*_2_) convention). Magneto-structural relationships in all doubly bridged [Cu_2_(OPh)(µ-1,1-N_3_)] core were examined. The effect of µ-phenolato and µ-azido bridge, and intermetallic distance (d) on the magnitude of coupling constant were investigated.

## Figures and Tables

**Table 1:** Magneto-chemical parameters for the µ-phenolate dicopper complexes with µ-azido as exogenous bridge.

Compound/Formula		Cu∙∙∙Cu [Å]	Cu–OPh–Cu [°]	Cu–N–Cu [°]	−J [cm^−1^]	Geometry	Ref
**1**	[Cu_4_(HL^2−^)_2_(µ-N_3_)_4_]	3.092	101.88	102.4	278	SP/SPI	TW
**2**	[Cu_2_(H_3_L1^2−^)(µ-N_3_)(H_2_O)(NO_3_)]	3.019	100.80	100.07	177.3	SP/SP	TW
**3**	{[Cu_2_(H_2_L2)(N_3_)_3_]•H_2_O•0.7CH_3_OH}*_n_*	3.112	102.3	104.09	512	SP/SP	[41]
**4**	{[Cu_2_(H_2_L2)(N_3_)_3_]•CH_3_OH}_n_	3.125	102.7	103.20	330	OC/OC	[41]
**5**	{[Cu_2_(HL2)(N_3_)]ClO_4_•0.8(CH_3_OH)}_n_	3.042	101.1	99.87	347	SPL/SP	[41]
**6**	{[Cu_2_L3(N_3_)_3_](ClO_4_) _2_}_n_	3.12	102.9	104.5	264	SP/SP	[119]
**7**	[Cu_2_(L4)(N_3_)(ClO_4_)_2_]•_n_H_2_O	2.972	98.7	100.0	86.5	OC/SP	[120]
**8**	[Cu_2_(L5)(N_3_)](ClO_4_)_2_•_n_H_2_O	2.993	100.5	99.7	161	OC/SP	[121]
**9**	[Cu_2_(L6)(μ-N_3_)(N_3_)_2_]•_n_H_2_O	3.032	101.3	98.26	188.6	OC/SP	[122]
**10**	[Cu_2_(L7)(N_3_)]	3.035	99.4	106.1	278	OC/OC	[123]
**11**	[Cu_2_(L8)(N_3_)]•nH_2_O	3.007	100.4	102.8	408	OC/OC	[123]
**12**	[Cu_2_(HL9)(µ-N_3_)(H_2_O)(C_2_H_5_OH) (C1O_4_) ]	2.968	99.2	99.9	66.2	OC/SP	[124]
**13**	[Cu_2_(L10)(N_3_)][PF_6_]_2_	3.185	107.9	103.6	440	SP/SP	[125]
**14**	[Cu_3_(L11)_2_(µ_1,1_-N_3_)_2_(CH_3_OH)_2_(BF_4_)_2_]	2.988	100.8	96.5	9.86	SP/OC	[114]
**15**	[Cu_3_(L11)_2_(µ_1,1_-N_3_)_2_(µ-NO_3_)_2_]	2.977	97.1	97.2	11.6	SP/OC	[114]
**16**	[Cu_3_(L12)_2_(µ_1,1_-N_3_)_2_(CH_3_OH)_2_(BF_4_)_2_]	2.996	97.1	97.4	19.98	SP/OC	[114]
**17**	[{Cu_2_ (L13)(µ1,1-N_3_)(ClO_4_)}_2_(µ1,3-N_3_)_2_]	3.097	102.36	101.39	119.5	SP/OC	[111]
**18**	[(CuL14)_2_(µ1,1N_3_)_2_Cu(H_2_O)]·CH_3_OH	3.0071	100.09	97.54	64.42	SP/SP	[118]
**19**	[(CuL15)_2_(µ1,1-N_3_)_2_Cu(H_2_O)]· CH_3_OH	2.950	98.05	94.53	9.60	SP/SP	[118]
**20**	[(CuL16)_2_(µ1,1-N_3_)_2_Cu(H_2_O)]· 2CH_3_OH	2.9398	98.03	95.34	4.54	SP/SP	[118]
**21**	[Cu_2_ (L17)(µ1,1-N_3_)]·2H_2_O	3.10	102.06	102.94	119	SP/SP	[126]

SP—Square pyramidal; OC—Octahedral; SPl—Square Planar.

**Table 2:** Summary of crystallographic data for compounds **1**–**6**.

Compound	1	2	3
Empirical formula	C_30_H_40_Cu_4_N_16_O_6_	C_30_H_44_Cu_4_N_4_O_10_	C_15_H_19_Cu_2_N_6_O_9_
M	974.94	874.85	554.44
Crystal System	Monoclinic	monoclinic	monoclinic
Space group	C2/c	C2/c	P2_1_/c
a/Å	27.504(8)	17.6107(10)	10.1607(6)
b/Å	22.357(8)	11.8828(7)	24.3239(14)
c/ Å	7.187(3)	22.8905(17)	8.2923(5)
α/ ˚		-	
β/ ˚	93.526(7)	109.0710(10)	94.2440(10)
γ/ ˚		-	
*V/*A^3^	4798(3)	4527.3(5)	2043.8(2)
ρ_calcd_(g cm^−3^)	1.35	1.284	1.802
*T/*K	190	190	190.15
*Z*	4	4	4
μ/mm^−1^	1.801	1.899	2.144
Crystal size (mm)	0.4 × 0.2 × 0.1	0.5 × 0.2 × 0.2	0.2 × 0.2 × 0.15
Reflections collected:			
Total	18,862	17,838	16,291
Unique	4253	4004	3610
*R_int_*,	0.063	0.0193	0.0299
Final *R*_1_, *wR*_2_	0.0712, 0.2090	0.0401, 0.12732	0.0265, 0.0691
**Compound**	**4**	**5**	**6**
Empirical formula	C_15_H_24_BCu_2_F_4_N_5_O_7_	C_72_H_71_Cu_7_N_5_O_27_	C_88_H_100_Cu_10_N_8_O_32_
M	600.28	1883.11	2417.5
Crystal System	monoclinic	monoclinic	triclinic
Space group	P2_1_/c	P2_1_/c	Pī
a/Å	12.730(4)	20.291(3)	12.835(13)
b/Å	8.228(3)	22.194(3)	15.38(2)
c/ Å	20.879(6)	33.076(3)	15.81(2)
α/ ˚		-	103.149(17)
β/ ˚	97.208(5)	116.575(2)	113.233(9)
γ/ ˚		-	100.144(14)
*V/*A^3^	2169.7(12)	8892(2)	2666(6)
ρ_calcd_(g cm^−3^)	1.838	1.407	1.506
*T/*K	190.15	190	190
*Z*	4	4	1
μ/mm^−1^	2.044	1.714	2.028
Crystal size (mm)	0.45 × 0.15 × 0.15	0.2 × 0.2 × 0.1	0.2 × 0.2 × 0.1
Reflections collected:			
Total	15,704	66,906	9178
Unique	3838	15737	9178
*R_int_*,	0.0676	0.0795	0.0873
Final *R*_1_, *wR*_2_	0.0630, 0.1710	0.0679, 0.1817	0.0998, 0.2402

R*_int_* = Σ│*F_0_*^2^ − *F_0_*^2^(mean)│/ Σ *F_0_*^2^, R_1_ = Σ[│*F_0_*│ − │*F_c_*│]/Σ│*F_0_*│, wR_2_ = [Σ[w(│*F_0_*│^2^ − │*F_c_*│^2^)^2^]/Σ[w(│*F_0_*│^2^)^2^]]^1/2^; R = Σ||Fo| − |Fc||/Σ|Fo|, Rw = [Σw (|Fo| − |Fc|)^2^/Σw Fo^2^]^1/2^.

## Supplementary Materials

The following are available online, Selected interatomic distances and angles are listed in the Tables S1–S6 and UV-vis spectroscopy. Figure S1 (Perspective view of crystal packing of **3** along the ab axis, showing the formation of a 2D-sheet structure). Figure S2 (Perspective view of a portion of 1D-single chain in **4** along the b-axis). Powder XRD patterns for **1**, **3** and **5** in Figures S3–S5. Combined Checkcif report and combined crystallographic data file.
