# Supplementary material for: Self-Assembly of Antiferromagnetically-Coupled Copper(II) Supramolecular Architectures with Diverse Structural Complexities
Source: Molecules. 2020 Nov 26;25(23):5549. doi: 10.3390/molecules25235549 (PMC7734591; doi:10.3390/molecules25235549)

checkCIF/PLATON report

Structure factors have been supplied for datablock(s) n79_08_15_2011a_0m_a, np004, np010_0m, np011, np03, twin00_a

THIS REPORT IS FOR GUIDANCE ONLY. IF USED AS PART OF A REVIEW PROCEDURE FOR PUBLICATION, IT SHOULD NOT REPLACE THE EXPERTISE OF AN EXPERIENCED CRYSTALLOGRAPHIC REFEREE.

No syntax errors found. CIF dictionary Interpreting this report

Datablock: np004

```
Bond precision:  C-C = 0.0142 A          Wavelength=0.71073

Cell:             a=27.504(8)     b=22.357(8)        c=7.817(3)
                  alpha=90        beta=93.526(7)     gamma=90
Temperature:      190 K
```

	Calculated	Reported
Volume	4798(3)	4798(3)
Space group	C 2/c	C 1 2/c 1
Hall group	-C 2yc	-C 2yc
Moiety formula	C30 H40 Cu4 N16 O6 [+ solvent]	C30 H40 Cu4 N16 O6
Sum formula	C30 H40 Cu4 N16 O6 [+ solvent]	C30 H40 Cu4 N16 O6
Mr	974.98	974.94
Dx,g cm-3	1.350	1.350
Z	4	4
Mu (mm-1)	1.801	1.801
F000	1984.0	1984.0
F000’	1989.58	
h,k,lmax	32,26,9	32,26,9
Nref	4260	4253
Tmin,Tmax	0.656,0.835	0.497,0.746
Tmin’	0.482	

```
Correction method= # Reported T Limits: Tmin=0.497 Tmax=0.746
AbsCorr = MULTI-SCAN

Data completeness= 0.998          Theta(max)= 25.050

R(reflections)= 0.0712( 2814)     wR2(reflections)= 0.2428( 4253)

S = 1.065                   Npar= 257
```

The following ALERTS were generated. Each ALERT has the format
test-name_ALERT_alert-type_alert-level.
Click on the hyperlinks for more details of the test.

Alert level B

```
PLAT220_ALERT_2_B Non-Solvent  Resd 1  C   Ueq(max)/Ueq(min) Range       7.1 Ratio
PLAT420_ALERT_2_B D-H Without Acceptor       O3       --H3       .     Please Check
```

Alert level C

```
DIFMX02_ALERT_1_C  The maximum difference density is > 0.1*ZMAX*0.75
            The relevant atom site should be identified.
PLAT094_ALERT_2_C Ratio of Maximum / Minimum Residual Density ....      3.49 Report
PLAT097_ALERT_2_C Large Reported Max.  (Positive) Residual Density      2.46 eA-3
PLAT213_ALERT_2_C Atom C11             has ADP max/min Ratio .....       3.5 prolat
PLAT220_ALERT_2_C Non-Solvent  Resd 1  N   Ueq(max)/Ueq(min) Range       4.2 Ratio
PLAT220_ALERT_2_C Non-Solvent  Resd 1  O   Ueq(max)/Ueq(min) Range       4.2 Ratio
PLAT222_ALERT_3_C Non-Solv.  Resd 1  H   Uiso(max)/Uiso(min) Range       6.9 Ratio
PLAT230_ALERT_2_C Hirshfeld Test Diff for    C9       --C10      .       5.7 s.u.
PLAT241_ALERT_2_C High   'MainMol' Ueq as Compared to Neighbors of       C10 Check
PLAT242_ALERT_2_C Low    'MainMol' Ueq as Compared to Neighbors of        N4 Check
PLAT242_ALERT_2_C Low    'MainMol' Ueq as Compared to Neighbors of        C9 Check
PLAT242_ALERT_2_C Low    'MainMol' Ueq as Compared to Neighbors of       C14 Check
PLAT341_ALERT_3_C Low Bond Precision on  C-C Bonds ...............   0.01423 Ang.
PLAT360_ALERT_2_C Short  C(sp3)-C(sp3) Bond  C9       - C10      .      1.36 Ang.
PLAT410_ALERT_2_C Short Intra H...H Contact  H9A      ..H10      .      1.97 Ang.
                                                      x,y,z  =     1_555 Check
PLAT905_ALERT_3_C Negative K value in the Analysis of Variance ...    -7.114 Report
PLAT911_ALERT_3_C Missing FCF Refl Between Thmin & STh/L=    0.596         7 Report
PLAT918_ALERT_3_C Reflection(s) with I(obs) much Smaller I(calc) .         3 Check
PLAT975_ALERT_2_C Check Calcd Resid. Dens.  1.08A   From O2             0.66 eA-3
PLAT976_ALERT_2_C Check Calcd Resid. Dens.  0.48A   From O2            -0.46 eA-3
PLAT978_ALERT_2_C Number C-C Bonds with Positive Residual Density.         0 Info
```

Alert level G

```
PLAT002_ALERT_2_G Number of Distance or Angle Restraints on AtSite         2 Note
PLAT003_ALERT_2_G Number of Uiso or Uij Restrained non-H Atoms ...         4 Report
PLAT007_ALERT_5_G Number of Unrefined Donor-H Atoms ..............         1 Report
PLAT072_ALERT_2_G SHELXL First  Parameter in WGHT  Unusually Large      0.14 Report
PLAT083_ALERT_2_G SHELXL Second Parameter in WGHT  Unusually Large     36.15 Why ?
PLAT172_ALERT_4_G The CIF-Embedded .res File Contains DFIX Records         1 Report
PLAT178_ALERT_4_G The CIF-Embedded .res File Contains SIMU Records         1 Report
PLAT343_ALERT_2_G Unusual sp3      Angle Range in Main Residue for       C10 Check
PLAT605_ALERT_4_G Largest Solvent Accessible VOID in the Structure       395 A**3
PLAT793_ALERT_4_G Model has Chirality at C10         (Centro SPGR)         R Verify
PLAT793_ALERT_4_G Model has Chirality at C14         (Centro SPGR)         S Verify
PLAT794_ALERT_5_G Tentative Bond Valency for Cu1       (II)      .      2.25 Info
PLAT860_ALERT_3_G Number of Least-Squares Restraints .............        19 Note
PLAT868_ALERT_4_G ALERTS Due to the Use of _smtbx_masks Suppressed         ! Info
PLAT909_ALERT_3_G Percentage of I>2sig(I) Data at Theta(Max) Still       40% Note
PLAT933_ALERT_2_G Number of OMIT Records in Embedded .res File ...         3 Note
```

0 **ALERT level A** = Most likely a serious problem - resolve or explain
2 **ALERT level B** = A potentially serious problem, consider carefully
21 **ALERT level C** = Check. Ensure it is not caused by an omission or oversight
16 **ALERT level G** = General information/check it is not something unexpected

```
 1 ALERT type 1 CIF construction/syntax error, inconsistent or missing data
23 ALERT type 2 Indicator that the structure model may be wrong or deficient
 7 ALERT type 3 Indicator that the structure quality may be low
 6 ALERT type 4 Improvement, methodology, query or suggestion
 2 ALERT type 5 Informative message, check
```

Datablock: np03

```
Bond precision:  C-C = 0.0039 A          Wavelength=0.71073

Cell:            a=10.1607(6)     b=24.3239(14)      c=8.2923(5)
                 alpha=90         beta=94.244(1)     gamma=90
Temperature:     190 K

                Calculated                Reported
Volume          2043.8(2)                 2043.8(2)
Space group     P 21/c                    P 1 21/c 1
Hall group      -P 2ybc                   -P 2ybc
Moiety formula  C15 H19 Cu2 N6 O9         C15 H19 Cu2 N6 O9
Sum formula     C15 H19 Cu2 N6 O9         C15 H19 Cu2 N6 O9
Mr              554.46                    554.44
Dx,g cm-3       1.802                     1.802
Z               4                         4
Mu (mm-1)       2.144                     2.144
F000            1124.0                    1124.0
F000'           1127.02
h,k,lmax        12,28,9                   12,28,9
Nref            3610                      3610
Tmin,Tmax       0.658,0.725               0.590,0.746
Tmin'           0.645

Correction method= # Reported T Limits: Tmin=0.590 Tmax=0.746
AbsCorr = MULTI-SCAN

Data completeness= 1.000          Theta(max)= 25.045

R(reflections)= 0.0265( 3175)     wR2(reflections)= 0.0722( 3610)

S = 1.025                  Npar= 309
```

The following ALERTS were generated. Each ALERT has the format
test-name_ALERT_alert-type_alert-level.
Click on the hyperlinks for more details of the test.

Alert level B

```
PLAT430_ALERT_2_B Short Inter D...A Contact  O2A      ..O5       .       2.78 Ang.
                                                   1+x,y,z  =      1_655 Check
```

Alert level C

```
PLAT094_ALERT_2_C Ratio of Maximum / Minimum Residual Density ....       2.14 Report
PLAT220_ALERT_2_C Non-Solvent  Resd 1  N   Ueq(max)/Ueq(min) Range        3.3 Ratio
PLAT230_ALERT_2_C Hirshfeld Test Diff for    N3       --N4       .        6.9 s.u.
PLAT309_ALERT_2_C Single Bonded Oxygen (C-O > 1.3 Ang) ..........         O2A Check
PLAT309_ALERT_2_C Single Bonded Oxygen (C-O > 1.3 Ang) ..........         O2B Check
PLAT430_ALERT_2_C Short Inter D...A Contact  O2A      ..O2B      .       2.87 Ang.
                                                 1-x,-y,2-z  =      3_657 Check
PLAT975_ALERT_2_C Check Calcd Resid. Dens.  1.03A   From C11             0.64 eA-3
```

Alert level G

```
PLAT007_ALERT_5_G Number of Unrefined Donor-H Atoms .............           2 Report
PLAT300_ALERT_4_G Atom Site Occupancy of O2A       Constrained at         0.5 Check
PLAT300_ALERT_4_G Atom Site Occupancy of O2B       Constrained at         0.5 Check
PLAT301_ALERT_3_G Main Residue  Disorder ..............(Resd  1  )         3% Note
PLAT367_ALERT_2_G Long?  C(sp?)-C(sp?) Bond  C9       - C11      .       1.52 Ang.
PLAT793_ALERT_4_G Model has Chirality at C9         (Centro SPGR)           R Verify
PLAT793_ALERT_4_G Model has Chirality at C13        (Centro SPGR)           S Verify
PLAT794_ALERT_5_G Tentative Bond Valency for Cu1      (II)       .       2.29 Info
PLAT794_ALERT_5_G Tentative Bond Valency for Cu2      (II)       .       2.29 Info
PLAT909_ALERT_3_G Percentage of I>2sig(I) Data at Theta(Max) Still        77% Note
PLAT910_ALERT_3_G Missing # of FCF Reflection(s) Below Theta(Min).          1 Note
PLAT933_ALERT_2_G Number of OMIT Records in Embedded .res File ...          1 Note
PLAT978_ALERT_2_G Number C-C Bonds with Positive Residual Density.          5 Info
```

```
   0 ALERT level A = Most likely a serious problem - resolve or explain
   1 ALERT level B = A potentially serious problem, consider carefully
   7 ALERT level C = Check. Ensure it is not caused by an omission or oversight
  13 ALERT level G = General information/check it is not something unexpected

   0 ALERT type 1 CIF construction/syntax error, inconsistent or missing data
  11 ALERT type 2 Indicator that the structure model may be wrong or deficient
   3 ALERT type 3 Indicator that the structure quality may be low
   4 ALERT type 4 Improvement, methodology, query or suggestion
   3 ALERT type 5 Informative message, check
```

Datablock: np010_0m

Bond precision:	C-C = 0.0085 A		Wavelength=0.71073
Cell:	a=12.730(4)	b=8.228(3)	c=20.879(6)
	alpha=90	beta=97.208(5)	gamma=90
Temperature:	190 K		

	Calculated	Reported
Volume	2169.6(12)	2169.7(12)
Space group	P 21/c	P 1 21/c 1
Hall group	-P 2ybc	-P 2ybc
Moiety formula	C15 H22 Cu2 N5 O6, B F4, H2 O	C15 H20 Cu2 N5 O5, B F4, 2(H2 O)
Sum formula	C15 H24 B Cu2 F4 N5 O7	C15 H24 B Cu2 F4 N5 O7
Mr	600.30	600.28
Dx,g cm-3	1.838	1.838
Z	4	4
Mu (mm-1)	2.044	2.044
F000	1216.0	1216.0
F000'	1219.20	
h,k,lmax	15,9,24	15,9,24
Nref	3837	3838
Tmin,Tmax	0.699,0.736	0.439,0.746
Tmin'	0.395	

Correction method= # Reported T Limits: Tmin=0.439 Tmax=0.746
AbsCorr = MULTI-SCAN

Data completeness= 1.000 Theta(max)= 25.049

R(reflections)= 0.0630(3011) wR2(reflections)= 0.1896(3838)

S = 1.051 Npar= 329

The following ALERTS were generated. Each ALERT has the format
test-name_ALERT_alert-type_alert-level.
Click on the hyperlinks for more details of the test.

Alert level B

```
PLAT417_ALERT_2_B Short Inter D-H..H-D       H6A      ..H7E      .       1.96 Ang.
                                                      x,y,z  =      1_555 Check
PLAT417_ALERT_2_B Short Inter D-H..H-D       H6B      ..H7D      .       2.09 Ang.
                                                 1-x,1-y,-z  =      3_665 Check
PLAT420_ALERT_2_B D-H Without Acceptor       O7       --H7D      .     Please Check
PLAT971_ALERT_2_B Check Calcd Resid. Dens.  1.07A   From C10             2.58 eA-3
```

Alert level C

```
DIFMX02_ALERT_1_C  The maximum difference density is > 0.1*ZMAX*0.75
            The relevant atom site should be identified.
PLAT094_ALERT_2_C Ratio of Maximum / Minimum Residual Density ....       2.68 Report
PLAT097_ALERT_2_C Large Reported Max.  (Positive) Residual Density       2.73 eA-3
PLAT222_ALERT_3_C Non-Solv.  Resd 1  H   Uiso(max)/Uiso(min) Range        6.3 Ratio
PLAT223_ALERT_4_C Solv./Anion  Resd 3  H   Ueq(max)/Ueq(min) Range        8.2 Ratio
PLAT230_ALERT_2_C Hirshfeld Test Diff for    O2       --C10      .        7.0 s.u.
PLAT232_ALERT_2_C Hirshfeld Test Diff (M-X)  Cu1      --O6       .        8.3 s.u.
PLAT245_ALERT_2_C U(iso) H7D       Smaller than U(eq) O7        by      0.034 Ang**2
PLAT250_ALERT_2_C Large U3/U1 Ratio for Average U(i,j) Tensor ....        2.5 Note
PLAT260_ALERT_2_C Large Average Ueq of Residue Including        F1      0.116 Check
```

```
PLAT341_ALERT_3_C Low Bond Precision on  C-C Bonds ...............    0.00846 Ang.
PLAT415_ALERT_2_C Short Inter D-H..H-X       H2       ..H8       .       2.13 Ang.
                                             -x,1/2+y,1/2-z  =      2_555 Check
PLAT417_ALERT_2_C Short Inter D-H..H-D       H6A      ..H7D      .       2.13 Ang.
                                                      x,y,z  =      1_555 Check
PLAT905_ALERT_3_C Negative K value in the Analysis of Variance ...     -1.057 Report
PLAT975_ALERT_2_C Check Calcd Resid. Dens.  0.92A   From O6              0.40 eA-3
PLAT976_ALERT_2_C Check Calcd Resid. Dens.  0.74A   From O2             -0.49 eA-3
PLAT976_ALERT_2_C Check Calcd Resid. Dens.  0.72A   From O2             -0.49 eA-3
PLAT977_ALERT_2_C Check Negative Difference Density on H2               -0.43 eA-3
PLAT977_ALERT_2_C Check Negative Difference Density on H3               -0.54 eA-3
PLAT978_ALERT_2_C Number C-C Bonds with Positive Residual Density.          0 Info
```

Alert level G

```
PLAT002_ALERT_2_G Number of Distance or Angle Restraints on AtSite         10 Note
PLAT004_ALERT_5_G Polymeric Structure Found with Maximum Dimension          1 Info
PLAT007_ALERT_5_G Number of Unrefined Donor-H Atoms ..............          2 Report
PLAT042_ALERT_1_G Calc. and Reported MoietyFormula Strings  Differ     Please Check
PLAT083_ALERT_2_G SHELXL Second Parameter in WGHT  Unusually Large      10.41 Why ?
PLAT172_ALERT_4_G The CIF-Embedded .res File Contains DFIX Records          5 Report
PLAT173_ALERT_4_G The CIF-Embedded .res File Contains DANG Records          2 Report
PLAT244_ALERT_4_G Low    'Solvent' Ueq as Compared to Neighbors of         B1 Check
PLAT794_ALERT_5_G Tentative Bond Valency for Cu1       (II)      .       2.26 Info
PLAT860_ALERT_3_G Number of Least-Squares Restraints .............         12 Note
PLAT909_ALERT_3_G Percentage of I>2sig(I) Data at Theta(Max) Still        64% Note
PLAT910_ALERT_3_G Missing # of FCF Reflection(s) Below Theta(Min).          1 Note
PLAT992_ALERT_5_G Repd & Actual _reflns_number_gt Values Differ by          1 Check
```

```
   0 ALERT level A = Most likely a serious problem - resolve or explain
   4 ALERT level B = A potentially serious problem, consider carefully
  20 ALERT level C = Check. Ensure it is not caused by an omission or oversight
  13 ALERT level G = General information/check it is not something unexpected

   2 ALERT type 1 CIF construction/syntax error, inconsistent or missing data
  21 ALERT type 2 Indicator that the structure model may be wrong or deficient
   6 ALERT type 3 Indicator that the structure quality may be low
   4 ALERT type 4 Improvement, methodology, query or suggestion
   4 ALERT type 5 Informative message, check
```

Datablock: n79_08_15_2011a_0m_a

```
Bond precision:  C-C = 0.0158 A          Wavelength=0.71073

Cell:              a=20.291(3)     b=22.194(3)          c=22.076(3)
                   alpha=90        beta=116.575(2)      gamma=90
Temperature:       190 K
```

	Calculated	Reported
Volume	8891(2)	8892(2)
Space group	P 21/c	P 1 21/c 1
Hall group	-P 2ybc	-P 2ybc
Moiety formula	C72 H71 Cu7 N4 O24, N O3 [+ solvent]	C72 H71 Cu7 N4 O24, N O3
Sum formula	C72 H71 Cu7 N5 O27 [+ solvent]	C72 H71 Cu7 N5 O27
Mr	1883.19	1883.11
Dx,g cm-3	1.407	1.407
Z	4	4
Mu (mm-1)	1.714	1.714
F000	3828.0	3828.0
F000’	3838.54	
h,k,lmax	24,26,26	24,26,26
Nref	15749	15737
Tmin,Tmax	0.717,0.842	0.629,0.745
Tmin’	0.703	

Correction method= # Reported T Limits: Tmin=0.629 Tmax=0.745
AbsCorr = MULTI-SCAN

Data completeness= 0.999 Theta(max)= 25.050

R(reflections)= 0.0670(9426) wR2(reflections)= 0.2264(15737)

S = 1.039 Npar= 1021

The following ALERTS were generated. Each ALERT has the format
test-name_ALERT_alert-type_alert-level.
Click on the hyperlinks for more details of the test.

Alert level B

```
PLAT213_ALERT_2_B Atom C14             has ADP max/min Ratio .....        4.8 prolat
PLAT230_ALERT_2_B Hirshfeld Test Diff for    O1       --C15      .       11.5 s.u.
PLAT241_ALERT_2_B High   'MainMol' Ueq as Compared to Neighbors of        C14 Check
PLAT241_ALERT_2_B High   'MainMol' Ueq as Compared to Neighbors of        C15 Check
PLAT242_ALERT_2_B Low    'MainMol' Ueq as Compared to Neighbors of        C13 Check
PLAT341_ALERT_3_B Low Bond Precision on  C-C Bonds ...............    0.01582 Ang.
PLAT420_ALERT_2_B D-H Without Acceptor       O2       --H2       .     Please Check
PLAT420_ALERT_2_B D-H Without Acceptor       O5       --H5       .     Please Check
PLAT420_ALERT_2_B D-H Without Acceptor       O6       --H6       .     Please Check
PLAT420_ALERT_2_B D-H Without Acceptor       O7       --H7       .     Please Check
PLAT420_ALERT_2_B D-H Without Acceptor       O10      --H10      .     Please Check
PLAT420_ALERT_2_B D-H Without Acceptor       O22      --H22      .     Please Check
PLAT430_ALERT_2_B Short Inter D...A Contact  O27      ..O27      .       2.70 Ang.
                                                 1-x,2-y,-z  =      3_675 Check
```

Alert level C

```
PLAT220_ALERT_2_C Non-Solvent  Resd 1  C   Ueq(max)/Ueq(min) Range        5.7 Ratio
PLAT220_ALERT_2_C Non-Solvent  Resd 1  O   Ueq(max)/Ueq(min) Range        4.4 Ratio
```

```
PLAT222_ALERT_3_C Non-Solv.  Resd 1  H   Uiso(max)/Uiso(min) Range        5.5 Ratio
PLAT230_ALERT_2_C Hirshfeld Test Diff for    O2       --C14      .        7.0 s.u.
PLAT234_ALERT_4_C Large Hirshfeld Difference C21      --C22      .       0.19 Ang.
PLAT241_ALERT_2_C High   'MainMol' Ueq as Compared to Neighbors of        O20 Check
PLAT241_ALERT_2_C High   'MainMol' Ueq as Compared to Neighbors of        C36 Check
PLAT241_ALERT_2_C High   'MainMol' Ueq as Compared to Neighbors of        C47 Check
PLAT241_ALERT_2_C High   'MainMol' Ueq as Compared to Neighbors of        C63 Check
PLAT242_ALERT_2_C Low    'MainMol' Ueq as Compared to Neighbors of        C31 Check
PLAT244_ALERT_4_C Low    'Solvent' Ueq as Compared to Neighbors of         N5 Check
PLAT260_ALERT_2_C Large Average Ueq of Residue Including       O25      0.182 Check
PLAT360_ALERT_2_C Short  C(sp3)-C(sp3) Bond  C13      - C14      .       1.38 Ang.
PLAT430_ALERT_2_C Short Inter D...A Contact  O26      ..O27      .       2.86 Ang.
                                                 1-x,2-y,-z  =      3_675 Check
PLAT905_ALERT_3_C Negative K value in the Analysis of Variance ...    -13.966 Report
PLAT905_ALERT_3_C Negative K value in the Analysis of Variance ...     -0.644 Report
PLAT910_ALERT_3_C Missing # of FCF Reflection(s) Below Theta(Min).          6 Note
PLAT911_ALERT_3_C Missing FCF Refl Between Thmin & STh/L=    0.596          7 Report
PLAT918_ALERT_3_C Reflection(s) with I(obs) much Smaller I(calc) .          1 Check
PLAT934_ALERT_3_C Number of (Iobs-Icalc)/Sigma(W) > 10 Outliers ..          1 Check
PLAT976_ALERT_2_C Check Calcd Resid. Dens.  1.02A   From O27            -0.63 eA-3
PLAT976_ALERT_2_C Check Calcd Resid. Dens.  0.89A   From O25            -0.45 eA-3
PLAT976_ALERT_2_C Check Calcd Resid. Dens.  0.70A   From O2             -0.45 eA-3
PLAT976_ALERT_2_C Check Calcd Resid. Dens.  0.83A   From O26            -0.43 eA-3
PLAT978_ALERT_2_C Number C-C Bonds with Positive Residual Density.          0 Info
```

Alert level G

```
PLAT002_ALERT_2_G Number of Distance or Angle Restraints on AtSite         15 Note
PLAT003_ALERT_2_G Number of Uiso or Uij Restrained non-H Atoms ...          7 Report
PLAT007_ALERT_5_G Number of Unrefined Donor-H Atoms .............           2 Report
PLAT072_ALERT_2_G SHELXL First  Parameter in WGHT  Unusually Large       0.12 Report
PLAT083_ALERT_2_G SHELXL Second Parameter in WGHT  Unusually Large      28.59 Why ?
PLAT172_ALERT_4_G The CIF-Embedded .res File Contains DFIX Records          5 Report
PLAT173_ALERT_4_G The CIF-Embedded .res File Contains DANG Records          6 Report
PLAT177_ALERT_4_G The CIF-Embedded .res File Contains DELU Records          3 Report
PLAT186_ALERT_4_G The CIF-Embedded .res File Contains ISOR Records          1 Report
PLAT432_ALERT_2_G Short Inter X...Y Contact  O27      ..N5               2.15 Ang.
                                                 1-x,2-y,-z  =      3_675 Check
PLAT432_ALERT_2_G Short Inter X...Y Contact  N5       ..N5               2.38 Ang.
                                                 1-x,2-y,-z  =      3_675 Check
PLAT606_ALERT_4_G VERY LARGE Solvent Accessible VOID(S) in Structure        ! Info
PLAT793_ALERT_4_G Model has Chirality at C9         (Centro SPGR)           S Verify
PLAT793_ALERT_4_G Model has Chirality at C13        (Centro SPGR)           S Verify
PLAT793_ALERT_4_G Model has Chirality at C60        (Centro SPGR)           S Verify
PLAT793_ALERT_4_G Model has Chirality at C64        (Centro SPGR)           S Verify
PLAT794_ALERT_5_G Tentative Bond Valency for Cu1      (II)      .        2.31 Info
PLAT794_ALERT_5_G Tentative Bond Valency for Cu2      (II)      .        2.29 Info
PLAT794_ALERT_5_G Tentative Bond Valency for Cu3      (II)      .        2.16 Info
PLAT794_ALERT_5_G Tentative Bond Valency for Cu4      (II)      .        2.16 Info
PLAT794_ALERT_5_G Tentative Bond Valency for Cu5      (III)     .        2.66 Info
PLAT794_ALERT_5_G Tentative Bond Valency for Cu6      (II)      .        2.33 Info
PLAT794_ALERT_5_G Tentative Bond Valency for Cu7      (II)      .        2.20 Info
PLAT860_ALERT_3_G Number of Least-Squares Restraints ............          32 Note
PLAT868_ALERT_4_G ALERTS Due to the Use of _smtbx_masks Suppressed          ! Info
PLAT909_ALERT_3_G Percentage of I>2sig(I) Data at Theta(Max) Still        31% Note
PLAT913_ALERT_3_G Missing # of Very Strong Reflections in FCF ....          1 Note
PLAT933_ALERT_2_G Number of OMIT Records in Embedded .res File ...          8 Note
```

---

0 **ALERT level A** = Most likely a serious problem - resolve or explain
13 **ALERT level B** = A potentially serious problem, consider carefully
25 **ALERT level C** = Check. Ensure it is not caused by an omission or oversight

28 **ALERT level G** = General information/check it is not something unexpected

0 ALERT type 1 CIF construction/syntax error, inconsistent or missing data
35 ALERT type 2 Indicator that the structure model may be wrong or deficient
11 ALERT type 3 Indicator that the structure quality may be low
12 ALERT type 4 Improvement, methodology, query or suggestion
8 ALERT type 5 Informative message, check

Datablock: twin00_a

Bond precision: C-C = 0.0265 A Wavelength=0.71073

Cell:	a=12.835(13)	b=15.38(2)	c=15.81(2)
	alpha=103.149(17)	beta=113.233(9)	gamma=100.144(14)
Temperature:	190 K		

	Calculated	Reported
Volume	2667(6)	2666(6)
Space group	P -1	P -1
Hall group	-P 1	-P 1
Moiety formula	C88 H100 Cu10 N8 O32 [+ solvent]	C88 H100 Cu10 N8 O32
Sum formula	C88 H100 Cu10 N8 O32 [+ solvent]	C88 H100 Cu10 N8 O32
Mr	2417.27	2417.15
Dx,g cm-3	1.505	1.506
Z	1	1
Mu (mm-1)	2.027	2.028
F000	1230.0	1230.0
F000’	1233.67	
h,k,lmax	15,18,18	0,0,0
Nref	9442	9178
Tmin,Tmax	0.673,0.816	0.008,0.027
Tmin’	0.660	

Correction method= # Reported T Limits: Tmin=0.008 Tmax=0.027
AbsCorr = MULTI-SCAN

Data completeness= 0.972 Theta(max)= 25.050

R(reflections)= 0.0996(3395) wR2(reflections)= 0.2739(9178)

S = 0.886 Npar= 622

The following ALERTS were generated. Each ALERT has the format
test-name_ALERT_alert-type_alert-level.
Click on the hyperlinks for more details of the test.

Alert level A

```
PLAT417_ALERT_2_A Short Inter D-H..H-D       H3       ..H5       .       1.43 Ang.
                                                -1+x,y,z  =      1_455 Check
```

Author Response: Hydrogen atoms for disordered hydroxyl groups containing O3 O5 were fixed. Efforts to relocate them were unsuccessful.

Alert level B

```
RINTA01_ALERT_3_B  The value of Rint is greater than 0.18
            Rint given   0.181
PLAT020_ALERT_3_B The Value of Rint is Greater Than 0.12 .........      0.181 Report
PLAT026_ALERT_3_B Ratio Observed / Unique Reflections (too) Low ..        37% Check
PLAT230_ALERT_2_B Hirshfeld Test Diff for    C13      --C15      .        7.5 s.u.
PLAT341_ALERT_3_B Low Bond Precision on  C-C Bonds ..............     0.0265 Ang.
PLAT416_ALERT_2_B Short Intra D-H..H-D       H4       ..H14      .       1.86 Ang.
                                                   x,y,z  =      1_555 Check
PLAT417_ALERT_2_B Short Inter D-H..H-D       H13      ..H15      .       1.83 Ang.
                                                -1+x,y,z  =      1_455 Check
```

Author Response: Hydrogen atoms for disordered hydroxyl groups containing O3 O5 were fixed. Efforts to relocate them were unsuccessful.

```
PLAT420_ALERT_2_B D-H Without Acceptor       O4       --H4       .     Please Check
PLAT420_ALERT_2_B D-H Without Acceptor       O13      --H13      .     Please Check
PLAT420_ALERT_2_B D-H Without Acceptor       O14      --H14      .     Please Check
PLAT919_ALERT_3_B Reflection # Likely Affected by the Beamstop ...          3 Check
PLAT934_ALERT_3_B Number of (Iobs-Icalc)/Sigma(W) > 10 Outliers ..          4 Check
```

Alert level C

```
PLAT029_ALERT_3_C _diffrn_measured_fraction_theta_full value Low .      0.972 Why?
PLAT084_ALERT_3_C High wR2 Value (i.e. > 0.25) ...................       0.27 Report
PLAT148_ALERT_3_C s.u. on the      a    - Axis is (Too) Large ....      0.013 Ang.
PLAT148_ALERT_3_C s.u. on the      b    - Axis is (Too) Large ....      0.020 Ang.
PLAT148_ALERT_3_C s.u. on the      c    - Axis is (Too) Large ....      0.020 Ang.
PLAT213_ALERT_2_C Atom C11             has ADP max/min Ratio .....        3.7 prolat
PLAT213_ALERT_2_C Atom C39             has ADP max/min Ratio .....        3.9 prolat
PLAT220_ALERT_2_C Non-Solvent  Resd 1  C   Ueq(max)/Ueq(min) Range        5.0 Ratio
PLAT220_ALERT_2_C Non-Solvent  Resd 1  O   Ueq(max)/Ueq(min) Range        4.2 Ratio
PLAT222_ALERT_3_C Non-Solv.  Resd 1  H   Uiso(max)/Uiso(min) Range        4.8 Ratio
PLAT230_ALERT_2_C Hirshfeld Test Diff for    O3       --C11      .        5.8 s.u.
PLAT234_ALERT_4_C Large Hirshfeld Difference O12      --C39      .       0.20 Ang.
PLAT234_ALERT_4_C Large Hirshfeld Difference N1       --C9       .       0.24 Ang.
PLAT234_ALERT_4_C Large Hirshfeld Difference C38      --C39      .       0.18 Ang.
PLAT234_ALERT_4_C Large Hirshfeld Difference C42      --C43      .       0.18 Ang.
PLAT241_ALERT_2_C High   'MainMol' Ueq as Compared to Neighbors of        O7 Check
PLAT241_ALERT_2_C High   'MainMol' Ueq as Compared to Neighbors of       C18 Check
PLAT241_ALERT_2_C High   'MainMol' Ueq as Compared to Neighbors of       C20 Check
PLAT242_ALERT_2_C Low    'MainMol' Ueq as Compared to Neighbors of       C17 Check
PLAT415_ALERT_2_C Short Inter D-H..H-X       H13      ..H38      .       2.14 Ang.
                                               -x,1-y,-z  =      2_565 Check
```

```
PLAT906_ALERT_3_C Large K Value in the Analysis of Variance ......     10.048 Check
PLAT906_ALERT_3_C Large K Value in the Analysis of Variance ......      3.466 Check
PLAT906_ALERT_3_C Large K Value in the Analysis of Variance ......      2.061 Check
PLAT911_ALERT_3_C Missing FCF Refl Between Thmin & STh/L=    0.596        265 Report
PLAT918_ALERT_3_C Reflection(s) with I(obs) much Smaller I(calc) .          3 Check
PLAT977_ALERT_2_C Check Negative Difference Density on H4               -0.55 eA-3
PLAT977_ALERT_2_C Check Negative Difference Density on H10A             -0.52 eA-3
PLAT977_ALERT_2_C Check Negative Difference Density on H10B             -0.33 eA-3
PLAT977_ALERT_2_C Check Negative Difference Density on H14              -0.33 eA-3
PLAT977_ALERT_2_C Check Negative Difference Density on H43B             -0.31 eA-3
PLAT978_ALERT_2_C Number C-C Bonds with Positive Residual Density.          0 Info
```

Alert level G

```
PLAT002_ALERT_2_G Number of Distance or Angle Restraints on AtSite         10 Note
PLAT003_ALERT_2_G Number of Uiso or Uij Restrained non-H Atoms ...          8 Report
PLAT007_ALERT_5_G Number of Unrefined Donor-H Atoms ..............          4 Report
PLAT072_ALERT_2_G SHELXL First  Parameter in WGHT  Unusually Large       0.13 Report
PLAT172_ALERT_4_G The CIF-Embedded .res File Contains DFIX Records          3 Report
PLAT173_ALERT_4_G The CIF-Embedded .res File Contains DANG Records          4 Report
PLAT186_ALERT_4_G The CIF-Embedded .res File Contains ISOR Records          1 Report
PLAT187_ALERT_4_G The CIF-Embedded .res File Contains RIGU Records          2 Report
PLAT606_ALERT_4_G VERY LARGE Solvent Accessible VOID(S) in Structure        ! Info
PLAT793_ALERT_4_G Model has Chirality at C9          (Centro SPGR)          R Verify
PLAT793_ALERT_4_G Model has Chirality at C13         (Centro SPGR)          R Verify
PLAT793_ALERT_4_G Model has Chirality at C38         (Centro SPGR)          R Verify
PLAT793_ALERT_4_G Model has Chirality at C42         (Centro SPGR)          R Verify
PLAT794_ALERT_5_G Tentative Bond Valency for Cu1       (II)      .       2.24 Info
PLAT794_ALERT_5_G Tentative Bond Valency for Cu2       (III)     .       2.69 Info
PLAT794_ALERT_5_G Tentative Bond Valency for Cu4       (II)      .       2.19 Info
PLAT794_ALERT_5_G Tentative Bond Valency for Cu5       (II)      .       2.27 Info
PLAT860_ALERT_3_G Number of Least-Squares Restraints .............         82 Note
PLAT868_ALERT_4_G ALERTS Due to the Use of _smtbx_masks Suppressed          ! Info
PLAT908_ALERT_2_G Max. Perc. Data with I > 2*s(I) per Res.Shell .      74.04% Note
PLAT961_ALERT_5_G Dataset Contains no Negative Intensities .......     Please Check
```

---

1 **ALERT level A** = Most likely a serious problem - resolve or explain
12 **ALERT level B** = A potentially serious problem, consider carefully
31 **ALERT level C** = Check. Ensure it is not caused by an omission or oversight
21 **ALERT level G** = General information/check it is not something unexpected

0 ALERT type 1 CIF construction/syntax error, inconsistent or missing data
27 ALERT type 2 Indicator that the structure model may be wrong or deficient
18 ALERT type 3 Indicator that the structure quality may be low
14 ALERT type 4 Improvement, methodology, query or suggestion
6 ALERT type 5 Informative message, check

Datablock: np011

```
Bond precision:  C-C = 0.0062 A          Wavelength=0.71073

Cell:            a=17.6107(10)    b=11.8828(7)       c=22.8905(17)
                 alpha=90         beta=109.071(1)    gamma=90
Temperature:     190 K
```

	Calculated	Reported
Volume	4527.3(5)	4527.3(5)
Space group	C 2/c	C 1 2/c 1
Hall group	-C 2yc	-C 2yc
Moiety formula	C30 H44 Cu4 N4 O10 [+ solvent]	C30 H44 Cu4 N4 O10
Sum formula	C30 H44 Cu4 N4 O10 [+ solvent]	C30 H44 Cu4 N4 O10
Mr	874.89	874.85
Dx,g cm-3	1.284	1.284
Z	4	4
Mu (mm-1)	1.899	1.899
F000	1792.0	1792.0
F000’	1797.72	
h,k,lmax	20,14,27	20,14,27
Nref	4015	4004
Tmin,Tmax	0.640,0.684	0.456,0.746
Tmin’	0.383	

Correction method= # Reported T Limits: Tmin=0.456 Tmax=0.746
AbsCorr = MULTI-SCAN

Data completeness= 0.997 Theta(max)= 25.047

R(reflections)= 0.0401(3738) wR2(reflections)= 0.1286(4004)

S = 1.165 Npar= 221

The following ALERTS were generated. Each ALERT has the format
test-name_ALERT_alert-type_alert-level.
Click on the hyperlinks for more details of the test.

Alert level B

```
PLAT420_ALERT_2_B D-H Without Acceptor       O3       --H3       .     Please Check
PLAT420_ALERT_2_B D-H Without Acceptor       O5       --H5A      .     Please Check
PLAT420_ALERT_2_B D-H Without Acceptor       O5       --H5B      .     Please Check
PLAT919_ALERT_3_B Reflection # Likely Affected by the Beamstop ...          1 Check
PLAT934_ALERT_3_B Number of (Iobs-Icalc)/Sigma(W) > 10 Outliers ..          3 Check
```

Alert level C

```
PLAT341_ALERT_3_C Low Bond Precision on  C-C Bonds ...............    0.00623 Ang.
PLAT415_ALERT_2_C Short Inter D-H..H-X       H5A      ..H11      .       2.12 Ang.
                                        1/2-x,1/2+y,1/2-z  =      4_555 Check
PLAT906_ALERT_3_C Large K Value in the Analysis of Variance ......      2.421 Check
PLAT911_ALERT_3_C Missing FCF Refl Between Thmin & STh/L=    0.596         11 Report
PLAT918_ALERT_3_C Reflection(s) with I(obs) much Smaller I(calc) .          1 Check
PLAT975_ALERT_2_C Check Calcd Resid. Dens.  0.97A    From O2             0.50 eA-3
PLAT975_ALERT_2_C Check Calcd Resid. Dens.  0.86A    From O4             0.48 eA-3
PLAT977_ALERT_2_C Check Negative Difference Density on H5A              -0.46 eA-3
PLAT978_ALERT_2_C Number C-C Bonds with Positive Residual Density.          0 Info
```

Alert level G

```
PLAT007_ALERT_5_G Number of Unrefined Donor-H Atoms ..............          3 Report
PLAT083_ALERT_2_G SHELXL Second Parameter in WGHT  Unusually Large      28.43 Why ?
PLAT605_ALERT_4_G Largest Solvent Accessible VOID in the Structure       1190 A**3
PLAT793_ALERT_4_G Model has Chirality at C11         (Centro SPGR)          R Verify
PLAT793_ALERT_4_G Model has Chirality at C14         (Centro SPGR)          S Verify
PLAT794_ALERT_5_G Tentative Bond Valency for Cu2       (II)      .       2.22 Info
PLAT868_ALERT_4_G ALERTS Due to the Use of _smtbx_masks Suppressed          ! Info
PLAT909_ALERT_3_G Percentage of I>2sig(I) Data at Theta(Max) Still        87% Note
PLAT910_ALERT_3_G Missing # of FCF Reflection(s) Below Theta(Min).          1 Note
PLAT933_ALERT_2_G Number of OMIT Records in Embedded .res File ...         11 Note
```

```
   0 ALERT level A = Most likely a serious problem - resolve or explain
   5 ALERT level B = A potentially serious problem, consider carefully
   9 ALERT level C = Check. Ensure it is not caused by an omission or oversight
  10 ALERT level G = General information/check it is not something unexpected

   0 ALERT type 1 CIF construction/syntax error, inconsistent or missing data
  10 ALERT type 2 Indicator that the structure model may be wrong or deficient
   8 ALERT type 3 Indicator that the structure quality may be low
   4 ALERT type 4 Improvement, methodology, query or suggestion
   2 ALERT type 5 Informative message, check
```

It is advisable to attempt to resolve as many as possible of the alerts in all categories. Often the minor alerts point to easily fixed oversights, errors and omissions in your CIF or refinement strategy, so attention to these fine details can be worthwhile. In order to resolve some of the more serious problems it may be necessary to carry out additional measurements or structure refinements. However, the purpose of your study may justify the reported deviations and the more serious of these should normally be commented upon in the discussion or experimental section of a paper or in the "special_details" fields of the CIF. checkCIF was carefully designed to identify outliers and unusual parameters, but every test has its limitations and alerts that are not important in a particular case may appear. Conversely, the absence of alerts does not guarantee there are no aspects of the results needing attention. It is up to the individual to critically assess their own results and, if necessary, seek expert advice.

Publication of your CIF in IUCr journals

A basic structural check has been run on your CIF. These basic checks will be run on all CIFs submitted for publication in IUCr journals (*Acta Crystallographica*, *Journal of Applied Crystallography*, *Journal of Synchrotron Radiation*); however, if you intend to submit to *Acta Crystallographica Section C* or *E* or *IUCrData*, you should make sure that full publication checks are run on the final version of your CIF prior to submission.

Publication of your CIF in other journals

Please refer to the *Notes for Authors* of the relevant journal for any special instructions relating to CIF submission.

PLATON version of 07/08/2019; check.def file version of 30/07/2019

Datablock np004 - ellipsoid plot

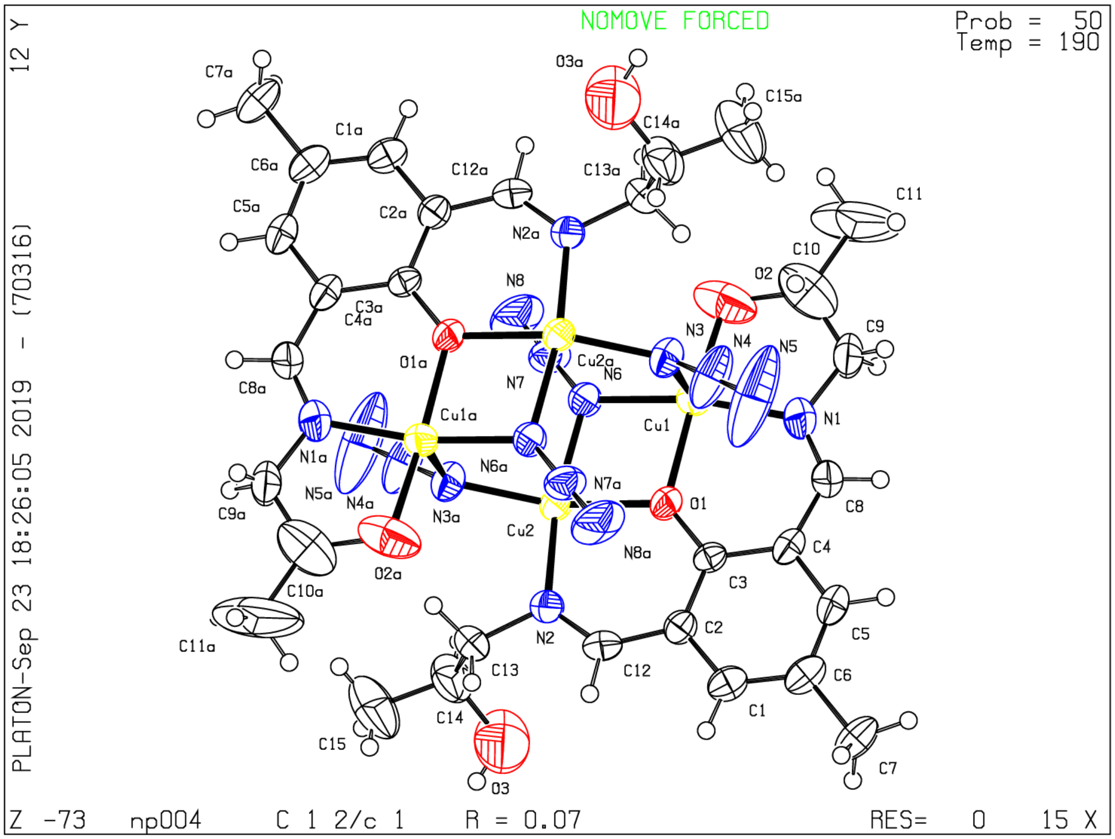

Datablock np03 - ellipsoid plot

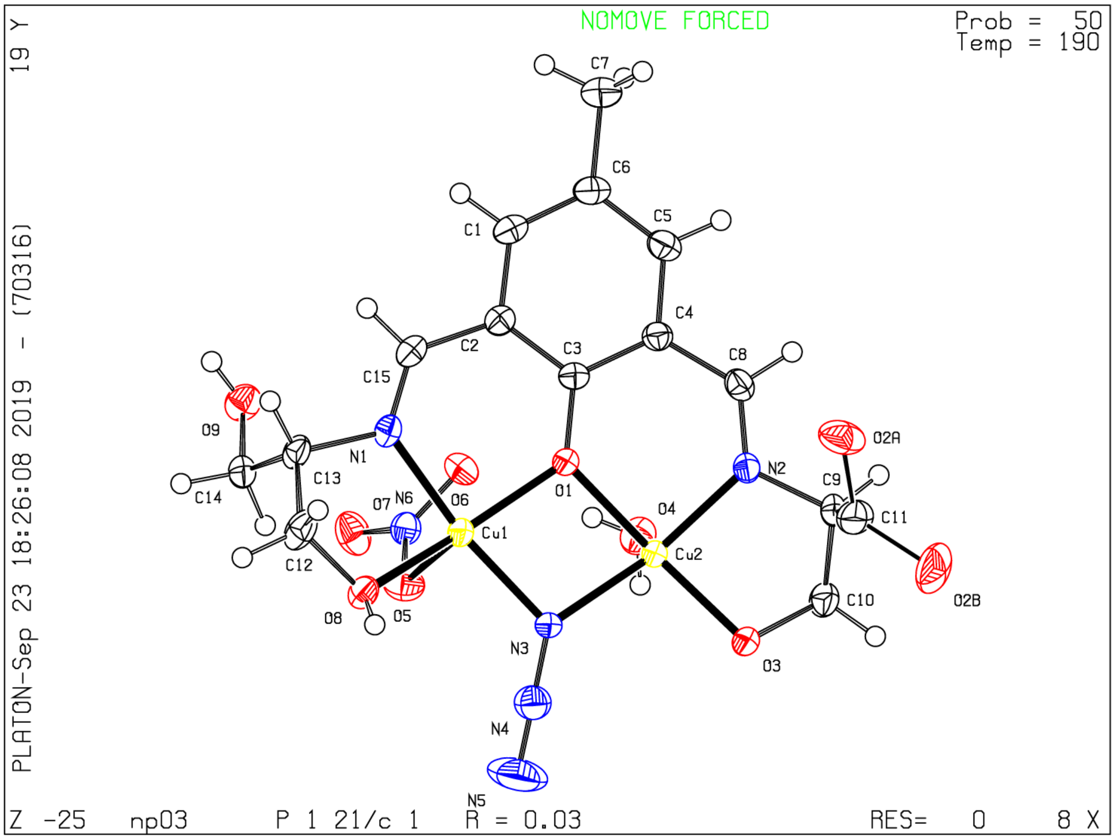

Datablock np010_0m - ellipsoid plot

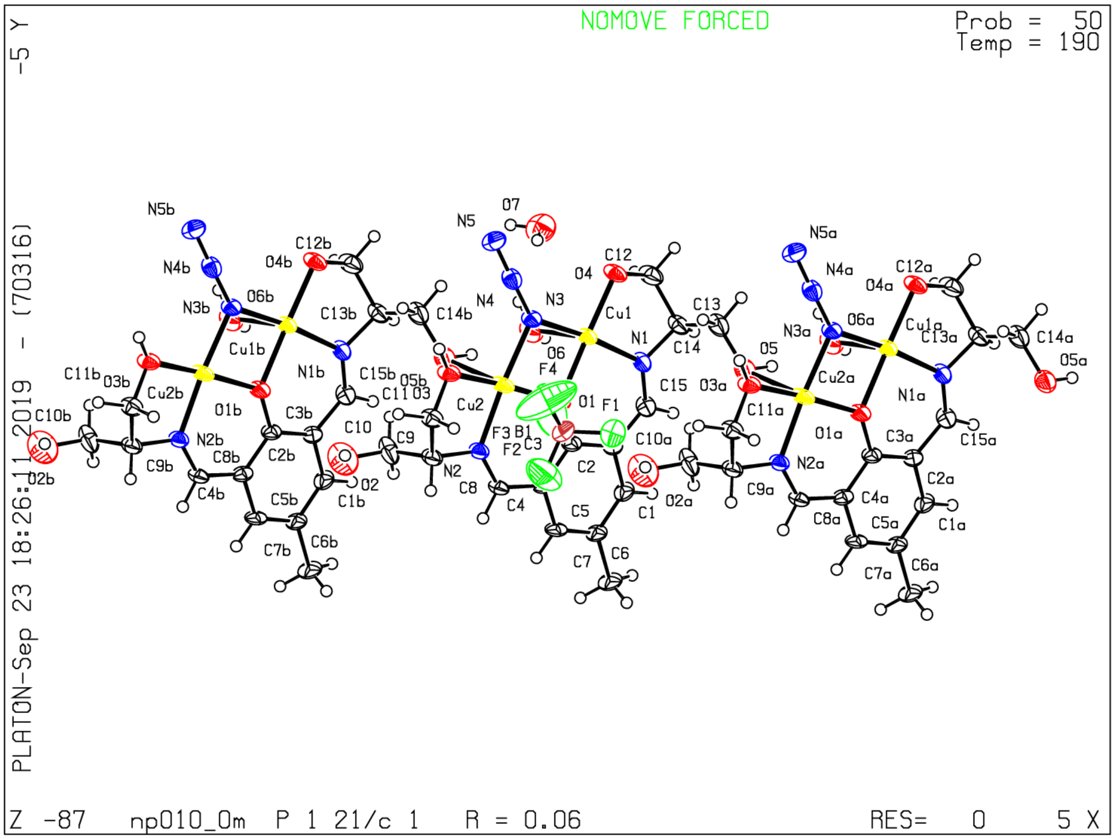

Datablock n79_08_15_2011a_0m_a - ellipsoid plot

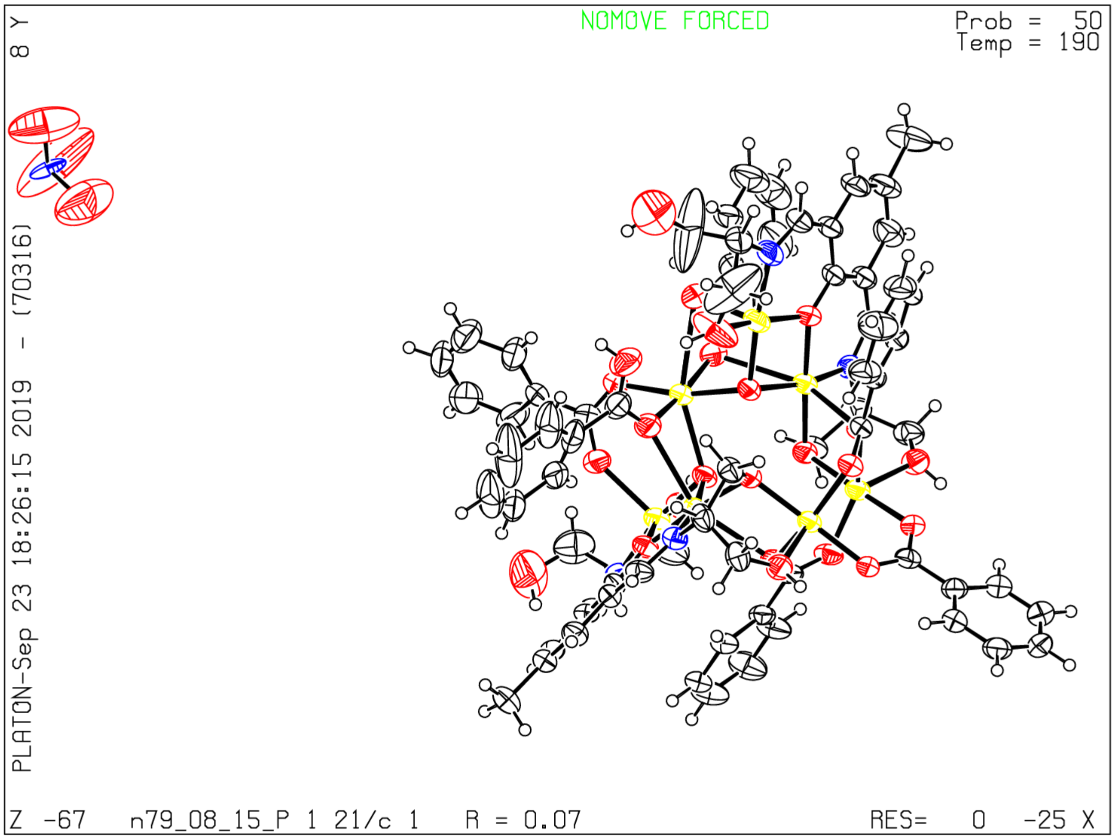

Datablock twin00_a - ellipsoid plot

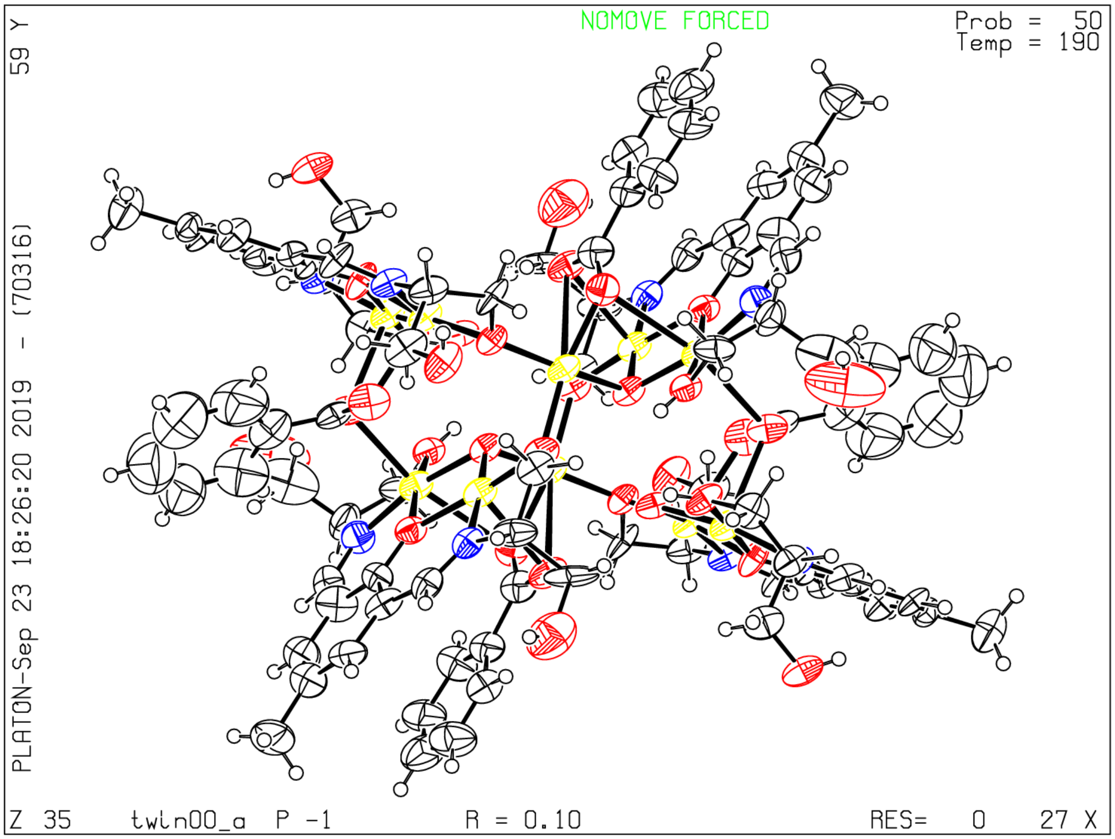

Datablock np011 - ellipsoid plot

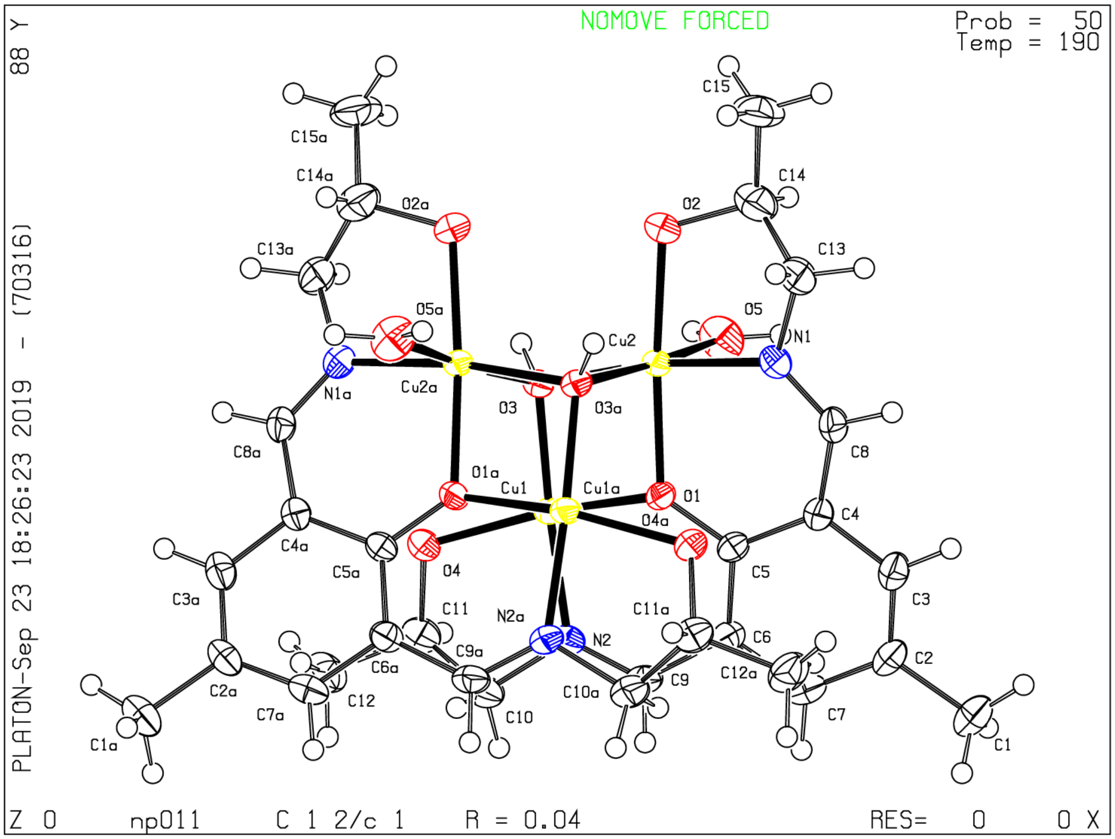

Supplement: Supplementary file 1 [file molecules-25-05549-s001.zip › molecules-994930-sup/checkcif_report.pdf]
