# Supplementary material for: Self-Assembly of Antiferromagnetically-Coupled Copper(II) Supramolecular Architectures with Diverse Structural Complexities
Source: Molecules. 2020 Nov 26;25(23):5549. doi: 10.3390/molecules25235549 (PMC7734591; doi:10.3390/molecules25235549)

Supplementary Information

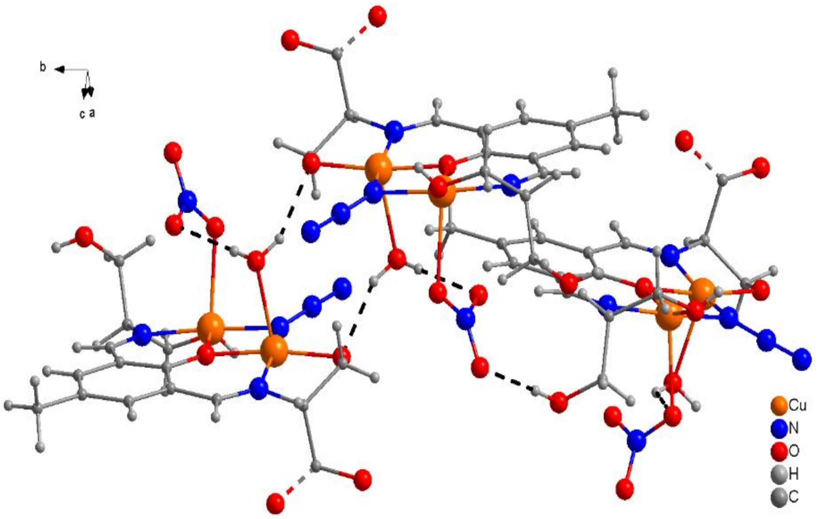

Figure S1. Perspective view of crystal packing of **3** along the *ab* axis, showing the formation of a 2D-sheet structure.

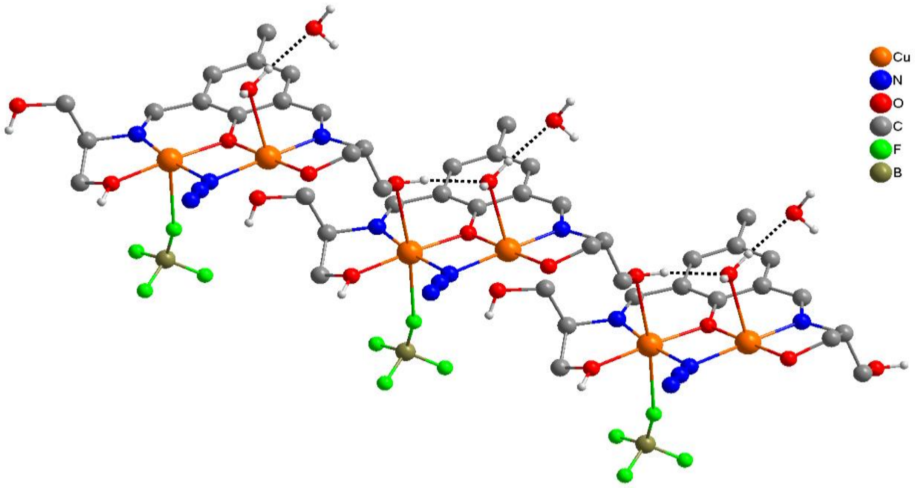

Figure S2. Perspective view of a portion of 1D-single chain in **4** along the *b*-axis.

Figures S3-S5 (XRD Pattern

Figures S3.

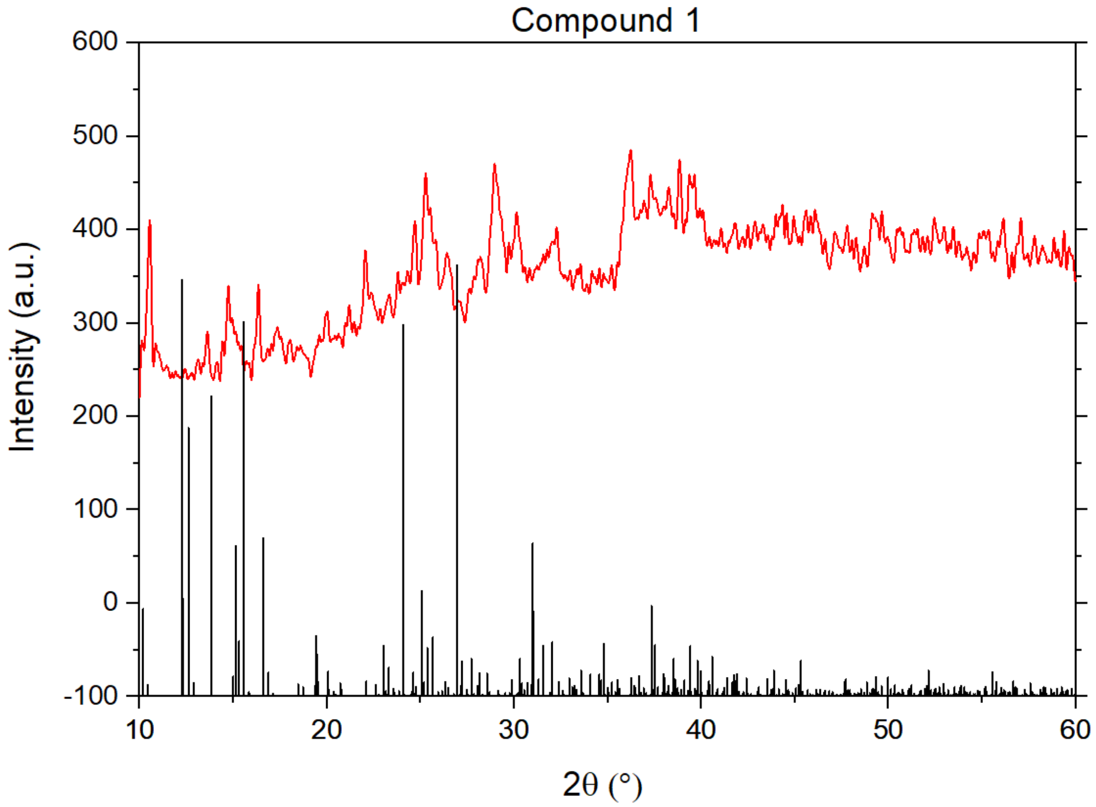

Figure S4.

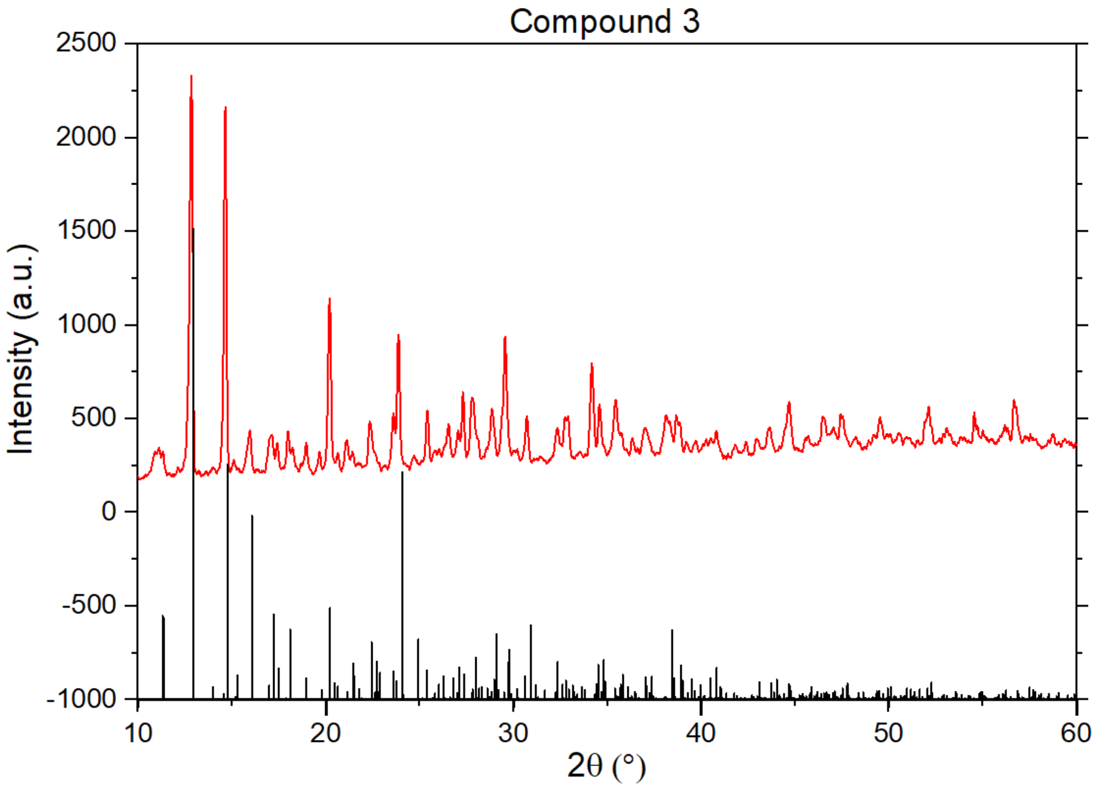

Figure S5.

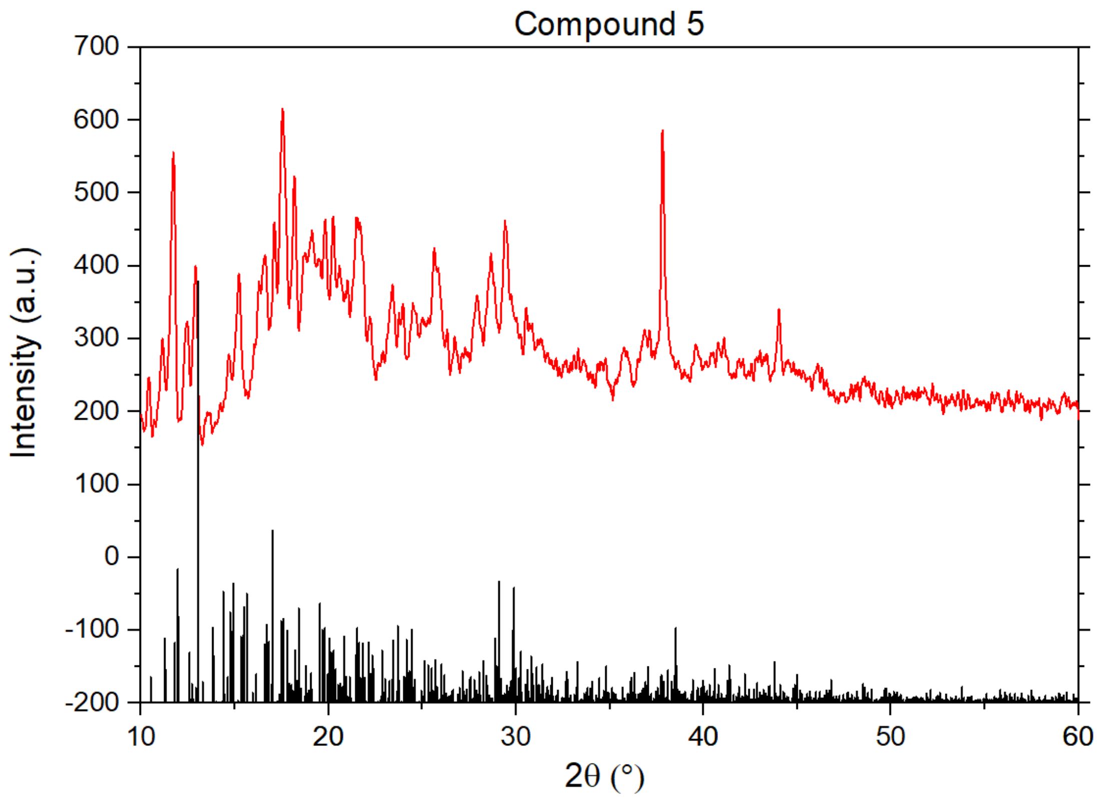

Electronic Supplementary Information

Tables S1-S6 for bond lengths and angles

Table S1. Bond lengths [Å] and angles [°] for **1**.

Bond lengths	[Å]	Bond angles	[°]
Cu(1)-O(1)	1.984(5)	N(1)-Cu(1)-O(1)	92.3(3)
Cu(1)-N(1)	1.944(7)	N(1)-Cu(1)-O(2)	85.6(4)
Cu(1)-N(6)	1.965(6)	O(2)-Cu(1)-N(6)	103.1(4)
Cu(1)-O(2)	1.943(7)	N6(1)-Cu(1)-O(1)	78.4(2)
Cu(1)-N(3)	2.422(7)	N(1)-Cu(1)-N(6)	169.4(3)
Cu(2)-O(1)	1.998(5)	O(1)-Cu(1)-O(2)	173.3(3)
Cu(2)-N(2)	1.959(6)	Cu(1)-O(1)-Cu(2)	101.88
Cu(2)-N(3)	1.968(7)	Cu(1)-N(6)-Cu(2)	102.4(3)
Cu(2)-N(6)	2.001(7)	N(2)-Cu(2)-O(1)	91.5(3)
Cu(1)-Cu(2)	3.092	N(2)-Cu(2)-N(3A)	95.1(3)
		N(3A)-Cu(2)-N(6)	94.4(3)
		N(6)-Cu(2)-O(1)	77.2(2)
		O(1)-Cu(2)-N(3A)	160.6(3)
		N(2)-Cu(2)-N(6)	167.6(3)
		Cu(1)-O(1)-Cu(2)	101.9(2)
		Cu(1)-N(3)-Cu(2A)	108.9(3)

Table S2. Bond lengths [Å] and angles [°] for **2.**

Bond lengths	[Å]	Bond angles	[°]
Cu(1)-O(1)	1.951(3)	O(1)-Cu(1)-O(4)	162.30(15)
Cu(1)-O(3)	1.944(3)	O(3)-Cu(1)-O(1)	81.52(14)
Cu(1)-O(4)	1.993(3)	O(3)-Cu(1)-O(4)	102.72(14)
Cu(1)-N(2)	1.938(4)	N2(1)-Cu(1)-O(1)	89.89(16)
Cu(2)-O(1)	1.979(3)	N(2)-Cu(1)-O(2)	168.32(17)
Cu(2)-O(2)	2.038(4)	N(2)-Cu(1)-O(4)	83.19(19)
Cu(2)-O(3)	1.956(3)	Cu(1)-O(1)-Cu(2)	98.02(14)
Cu(2)-O(3A)	2.360(3)	Cu(1)-O(3)-Cu(2)	92.05(16)
Cu(2)-O(5)	2.393(4)	Cu(1)-O(3)-Cu((2A)	110.07(15)
Cu(2)-N(1)	1.936(4)	Cu(2)-O(3)-Cu(2A)	95.07(13)
Cu(1)-Cu(2)	2.967	O(3)-Cu(2)-O(1)	80.54(13)
		O(3)-Cu(2)-O(2)	105.09(14)
		N(1)-Cu(2)-O(1)	91.00(16)
		N(1)-Cu(2)-O(2)	83.35(16)
		O(1)-Cu(2)-O(2)	174.08(14)
		N(1)-Cu(2)-O(3)	171.53(16)
		O(3A)-Cu(2)-O(5)	173.61(13)

Table S3. Bond distances [Å] and angles [°] for **3**.

Bond lengths	Å	Bond angles	[°]
Cu(1)-O(1)	1.9506(17)	N(1)-Cu(1)-O(1)	92.15(8)
Cu(1)-N(1)	1.915(2)	N(1)-Cu(1)-O(8)	84.67(9)
Cu(1)-N(3)	1.959(2)	O(8)-Cu(1)-N(3)	102.28(8)
Cu(1)-O(8)	1.9786(19)	N(3)-Cu(1)-O(1)	79.98(8)
Cu(1)-O(5)	2.384(2)	N(1)-Cu(1)-N(3)	172.06(8)
Cu(2)-O(1)	1.9680(17)	O(1)-Cu(1)-O(8)	158.96(8)
Cu(2)-N(2)	1.928(2)	N(2)-Cu(2)-O(1)	90.76(8)
Cu(2)-N(3)	1.980(2)	N(2)-Cu(2)-O(3)	86.84(8)
Cu(2)-O(3)	1.9341(17)	O(3)-Cu(2)-N(3)	102.02(8)
Cu(2)-O(4)	2.337(2)	N(3)-Cu(2)-O(1)	79.06(8)
Cu(1)-Cu(2)	3.019	O(1)-Cu(2)-O(3)	173.03(7)
		N(2)-Cu(2)-N(3)	165.33(9)
		Cu(1)-O(1)-Cu(2)	100.80(8)
		Cu(1)-N(3)-Cu(2)	100.07(9)

Table S4. Bond distances [Å] and angles [°] for **4.**

Bond lengths	Å	Bond angles	[°]
Cu(1)-O(1)	1.943(4)	N(1)-Cu(1)-O(1)	92.60(18)
Cu(1)-N(1)	1.928(5)	N(1)-Cu(1)-O(4)	86.10(19)
Cu(1)-N(3)	1.992(4)	O(4)-Cu(1)-N(3)	100.80(18)
Cu(1)-O(4)	1.916(4)	N(3)-Cu(1)-O(1)	80.47(18)

Cu(1)-O(6)	2.505(6)	N(1)-Cu(1)-N(3)	171.3(2)
Cu(2)-O(1)	1.941(4)	O(1)-Cu(1)-O(4)	178.67(18)
Cu(2)-N(2)	1.920(4)	N(2)-Cu(2)-O(1)	92.28(18)
Cu(2)-N(3)	1.971(5)	N(2)-Cu(2)-O(3)	85.10(18)
Cu(2)-O(3)	1.929(4)	O(3)-Cu(2)-N(3)	101.84(18)
Cu(2)-O(5)	2.797(4)	N(3)-Cu(2)-O(1)	81.02(17)
Cu(4)-F(4)	2.728(3)	O(1)-Cu(2)-O(3)	173.05(19)
Cu(1)-Cu(2)	2.985	N(2)-Cu(2)-N(3)	172.86(19)
		Cu(1)-O(1)-Cu(2)	100.44(17)
		Cu(1)-N(3)-Cu(2)	97.8(2)

Table S5. Bond distances [Å] and angles [°] for **5.**

Bond lengths	Å	Bond angles	[°]
Cu(4)-Cu(5)	2.8650(13)	O(4)-Cu(4)-O(15)	89.1(2)
Cu(4)-O(4)	1.940(5)	O(4)-Cu(4)-O(11)	85.9(2)
Cu(4)-O(5)	2.162(5)	O(5)-Cu(4)-Cu(5)	160.28(17)
Cu(4)-O(13)	1.920(5)	O(13)-Cu(4)-O(4)	176.2(2)
Cu(4)-O(15)	1.967(5)	O(13)-Cu(4)-O(15)	91.9(2)
Cu(4)-O(11)	2.004(5)	O(13)-Cu(4)-O(11)	94.8(2)
Cu(5)-O(20)	1.948(5)	O(15)-Cu(4)-O(11)	154.6(2)
Cu(5)-O(12)	1.984(5)	O(20)-Cu(5)-O(12)	88.1(2)
Cu(5)-O(16)	1.983(6)	O(20)-Cu(5)-O(16)	87.8(2)
Cu(5)-O(14)	1.920(5)	O(16)- Cu(5)-O(12)	156.8(2)
Cu(5)-O(21)	2.163(5)	O(14)-Cu(5)-O(20)	177.4(2)
Cu(7)-Cu(6)	2.9428(15)	O(14)-Cu(5)-O(12)	91.3(2)
Cu(7)-O(22)	1.933(5)	O(14)-Cu(5)-O(16)	93.7(2)
Cu(7)-O(17)	1.934(5)	O(21)-Cu(5)-Cu(4)	163.60(16)
Cu(7)-O(18)	1.975(6)	O(22)-Cu(7)-O(17)	81.2(2)
Cu(7)-O(10)	2.174(6)	O(22)-Cu(7)-O(18)	99.7(2)
Cu(7)-N(4)	1.935(7)	O(22)-Cu(7)-N(4)	166.3(2)
Cu(6)-O(20)	1.939(5)	O(17)-Cu(7)-O(18)	164.2(2)
Cu(6)-O(22)	1.966(5)	O(17)- Cu(7)-N(4)	91.1(2)
Cu(6)-O(17)	1.916(5)	N(4)-Cu(7)-O(18)	84.6(3)
Cu(6)-N(3)	1.914(6)	O(20)-Cu(6)-O(22)	101.9(2)
Cu(6)-O(23)	2.509(6)	O(17)-Cu(6)-O(20)	174.2(2)
Cu(6)-O(16)	2.483(5)	O(17)-Cu(6)-O(22)	80.8(2)
Cu(2)-Cu(1)	2.9389(15)	N(3)-Cu(6)-O(20)	85.0(2)
Cu(2)-O(4)	1.930(5)	N(3)-Cu(6)-O(22)	170.6(3)
Cu(2)-O(6)	1.983(5)	N(3)-Cu(6)-O(17)	92.9(2)
Cu(2)-O(1)	1.920(5)	O(4)-Cu(2)-O(6)	100.7(2)
Cu(2)-O(11)	2.363(6)	O(1)-Cu(2)-O(4)	177.1(2)
Cu(2)-N(2)	1.901(6)	O(1)-Cu(2)-O(6)	81.3(2)
Cu(3)-O(22)	2.258(5)	N(2)-Cu(2)-O(4)	86.2(2)

Cu(3)-O(6)	2.012(5)	N(2)-Cu(2)-O6	167.1(2)
Cu(3)-O(7)	1.995(6)	N(2)-Cu(2)-O(1)	92.3(3)
Cu(3)-O(23)	1.964(6)	O(7)-Cu(2)-O(11)	156.4(6)
Cu(3)-O(9)	1.919(6)	O(7)-Cu(3)-O(6)	86.9(2)
Cu(3)-O(8)	2.592(6)	O(23)-Cu(3)-O(6)	92.5(2)
Cu(1)-O(6)	1.945(5)	O(23)-Cu(3)-O(7)	172.5(2)
Cu(1)-O(1)	1.926(5)	O(9)-Cu(3)-O(6)	167.4(2)
Cu(1)-O(8)	2.382(7)	O(9)-Cu(3)-O(7)	88.7(2)
Cu(1)-N(1)	1.915(7)	O(9)-Cu(3)-O(23)	90.3(3)
Cu(1)-O(2)	1.928(7)	O(8-Cu(3)-O(22)	154.8(4)
		O(1)-Cu(1)-O(6)	82.1(2)
		O(1)-Cu(1)-O(2)	168.9(4)
		N(1)-Cu(1)-O(6)	174.8(3)
		N(1)-Cu(1)-O(1)	92.9(3)
		N(1)-Cu(1)-O(2)	86.1(3)
		O(2)-Cu(1)-O(6)	98.6(3)
		Cu(7)-O(22)-Cu(6)	98.0(2)
		Cu(7)-O(22)-Cu(3)	117.3(2)
		Cu(6)-O(22)-Cu(3)	102.1(2)
		Cu(6)-O(17)-Cu(7)	99.7(2)
		C(68)-O(17)-Cu(7)	130.6(5)
		C(68)-O(17)-Cu(6)	129.5(5)
		Cu(2)-O(6)-Cu(3)	109.3(3)
		Cu(1)-O(6)-Cu(2)	96.9(2)
		Cu(1)-O(6)-Cu(3)	109.6(2)
		Cu(2)-O(1)-Cu(1)	99.7(2)
		C(5)-O(1)-Cu(2)	130.8(6)
		C(5)-O(1)-Cu(1)	128.8(5)

Table S6. Bond distances [Å] and angles [°] for **6.**

Bond lengths	Å	Bond angles	[°]
Cu(2)-Cu(3[1])	3.004(4)	O(4)-Cu(2)-O(1)	171.6(4)
Cu(2)-O(8)	2.373(10)	O(6)-Cu(2)-O(1)	83.7(4)
Cu(2)-O(1)	1.938(10)	O(6)-Cu(2)-O(4)	95.8(4)
Cu(2)-O(4)	1.923(10)	N(2)-Cu(2)-O(1)	91.6(5)
Cu(2)-O(6)	1.920(10)	N(2)-Cu(2)-O(4)	87.2(5)
Cu(2)-N(2)	1.888(13)	N(2)-Cu(2)-O(6)	167.8(4)
Cu(2)-O(9)	2.59(2)	Cu(8)-Cu(2)-O(9)	165.2(3)
Cu(1)-O(1)	1.952(10)	O(1)-Cu(1)-O(6)	81.7(4)
Cu(1)-O(2)	1.874(10)	O(2)-Cu(1)-O(1)	174.5(4)
Cu(1)-O(6)	1.982(9)	O(2)-Cu(1)-O(6)	100.3(4)
Cu(1)-N(1)	1.912(12)	O(2)-Cu(1)-N(1)	87.8(5)
Cu(1)-O(10)	2.55(6)	N(1)-Cu(1)-O(1)	90.4(5)

Cu(3)-O(2)	1.931(9)	N(1)-Cu(1)-O(6)	171.6(5)
Cu(3)-O(6^1)	1.947(9)	O(2)-Cu(3)-O(6^1)	93.3(4)
Cu(3)-O(12)	1.897(9)	O(2)-Cu(3)-O(9)	162.7(4)
Cu(3)-O(9)	1.948(10)	O(6^1)-Cu(3)-O(9)	86.9(4)
Cu(5)-O(7)	2.243(12)	O(12)-Cu(3)-O(2)	92.1(4)
Cu(5)-O(16)	1.954(10)	O(12)-Cu(3)-O(6^1)	165.8(4)
Cu(5)- O(11)	1.952(9)	O(12)-Cu(3)-O(9)	91.8(4)
Cu(5)-O(12)	1.936(9)	O(11)-Cu(5)-O(16)	81.5(4)
Cu(5)-N(4)	1.932(12)	O(12)-Cu(5)-O(16)	101.2(4)
Cu(4)-O(8)	2.403(10)	O(12)-Cu(5)-O(11)	171.6(4)
Cu(4)-O(16)	1.953(9)	N(4)-Cu(5)-O(16)	167.7(4)
Cu(4)-O(11)	1.956(11)	N(4)-Cu(5)-O(11)	90.7(5)
Cu(4)-O(14)	1.944(10)	N(4)-Cu(5)-O(12)	85.3(5)
Cu(4)-N(3)	1.934(11)	O(16)-Cu(4)-O(11)	81.4(4)
		O(14)-Cu(4)-O(16)	102.6(4)
		O(14)-Cu(4)-O(11)	174.5(4)
		N(3)-Cu(4)-O(16)	167.7(4)
		N(3)-Cu(4)-O(11)	89.6(5)
		N(3)-Cu(4)-O(14)	85.9(5)
		Cu(2)-O(8)-Cu(4)	106.6(4)
		C(16)-O(8)-Cu(2)	113.8(9)
		C(16)-O(8)-Cu(4)	116.4(9)
		Cu(2)-O(1)-Cu(1)	97.5(4)
		C(5)-O(1)-Cu(2)	129.7(10)
		C(5)-O(1)-Cu(1)	131.9(10)
		Cu(1)-O(2)-Cu(3)	123.5(5)
		C(10)-O(2)-Cu(1)	108.9(8)
		C(10)-O(2)-Cu(3)	113.6(9)
		Cu(2)-O(6)-Cu(1)	97.1(4)
		Cu(2)-O(6)-Cu(3^1)	102.0(5)
		Cu(3^1)-O(6)-Cu(1)	118.9(4)
		Cu(4)-O(16)-Cu(5)	97.4(4)
		Cu(5)-O(11)-Cu(4)	97.3(4)
		C(34)-O(11)-Cu(5)	128.0(9)
		C(34)-O(11)-Cu(4)	128.0(10)
		Cu(3)-O(12)-Cu(5)	117.6(5)
		C(39)-O(12)-Cu(3)	122.8(8)
		C(39)-O(12)-Cu(5)	106.5(7)

IR and UV-Vis Spectroscopy

In the IR spectrum (experimental section) of **1** which has EO intradimer and interdimer (μ-1,1-N_3) bridging azide groups, and **3**, **4**, and **7** which have only intradimer μ-1,1-N_3 ion, one, two, or three strong bands are observed at (cm^{-1}) 2093, 2037(**1**), 2110, 2074, 2050 (**3**), 2114, 2080 (**4**) and 2035 cm^{-1} (**7**), typical for such bridging moieties.[40,48,49] The IR spectra of **1**-**6** show one to three bands in the regions 3137-3423 cm^{-1} and two bands in the region 1608-1645 cm^{-1} due to υ(OH) (H_2O and CH_3OH) or υ(NH_2) and υ(C=N)/υ(C=O) of coordinated imine/carbonyl of benzoate groups respectively.

The UV-Vis spectra of **1**-**6** compounds, recorded in DMF solution, are characterized by a very strong band in the region 320-378 nm and a low energy shoulder in the region 370-420 nm, which are assigned to metal-azide/metal-ligand charge transfer transitions. The d-d transition, characteristic for Cu(II) is observed as a broad band in the region 620-665 nm, typical for square

pyramidal/octahedrally-based geometry.[67] A typical UV-Vis spectrum of complex **5** is shown in Fig, S1

Fig S1. UV-Vis spectrum of **5** in DMF

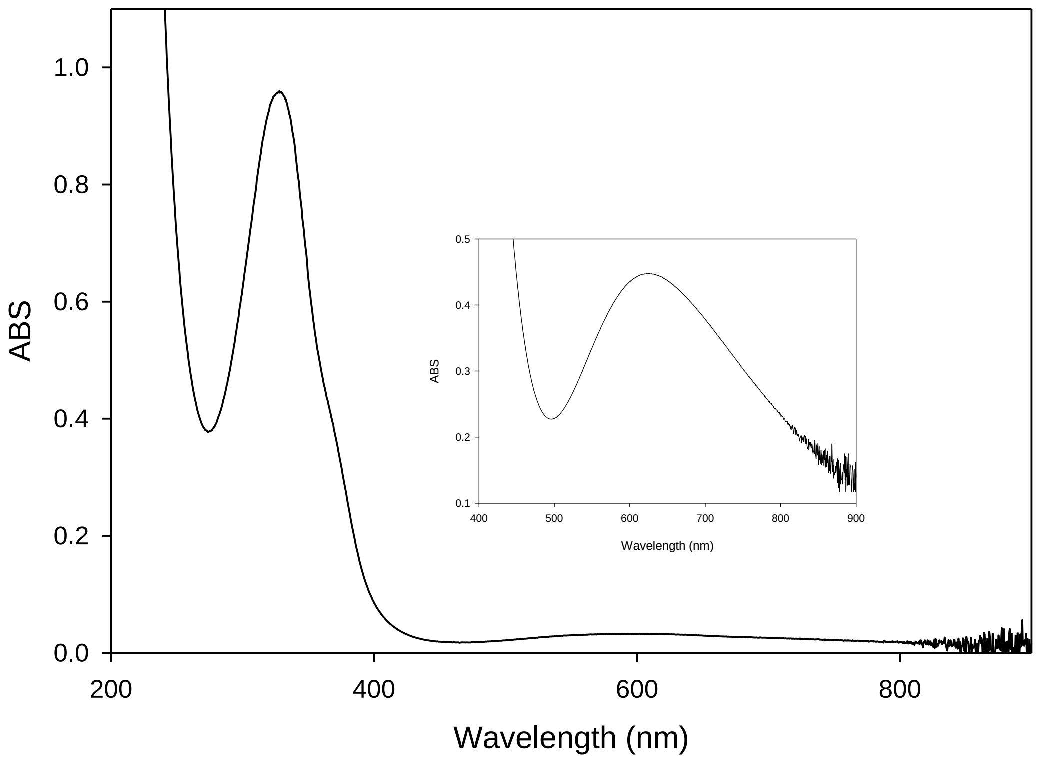

Supplement: Supplementary file 1 [file molecules-25-05549-s001.zip › molecules-994930-sup/molecules-994930-sup.pdf]
